# Symptom burden to characterise, predict, and prevent asthma attacks: a patient-level meta-analysis of randomised trials and translational prospective studies

**DOI:** 10.1016/S2213-2600(26)00150-5

**Published:** 2026-10

**Authors:** Simon Couillard, Fleur L Meulmeester, Pascal Charland, Samuel Lemaire-Paquette, Imran Howell, Carlos Celis-Preciado, Philippe Lachapelle, Liam G Heaney, Peter Bradding, Samuel Mailhot-Larouche, Sanjay Ramakrishnan, Michael E Wechsler, Guy Brusselle, Sarah E Diver, Christopher E Brightling, Mario Castro, Nicola A Hanania, David J Jackson, Neil Martin, Alison Moore, Peter Howarth, Megan E Hardin, Rebecca Gall, Cecile T J Holweg, Vennila Dharman, Richard W Beasley, Jacob K Sont, Ewout W Steyerberg, Ian D Pavord, The Oxford Asthma Attack Risk Scale Meta-analysis (ORACLE2) Consortium

**Affiliations:** a Faculté de médecine et des sciences de la santé, Université de Sherbrooke, Sherbrooke, QC, Canada; b Department of Biomedical Data Sciences, Caecilia Institute for Population Health and Data Sciences, Leiden University Medical Center, Leiden, Netherlands; c Respiratory Medicine Unit and Oxford Respiratory NIHR BRC, Nuffield Department of Medicine, University of Oxford, Oxford, UK; d Centre de recherche du CHUS, CIUSSS de l’Estrie – CHUS, Sherbrooke, QC, Canada; e Pulmonary Unit, Internal Medicine Department, Hospital Universitario San Ignacio, Bogota, Colombia; f Centre for Experimental Medicine, Queen's University Belfast, Belfast, UK; g Department of Respiratory Sciences, Institute for Lung Health, NIHR Respiratory BRC, University of Leicester, Leicester, UK; h Department of Respiratory Medicine, Sir Charles Gairdner Hospital, Perth, WA, Australia; i Institute for Respiratory Health and UWA Medical School, University of Western Australia, Perth, WA, Australia; j National Jewish Health, Denver, CO, USA; k Department of Respiratory Medicine, Ghent University Hospital, Ghent, Belgium; l Pulmonary, Critical Care and Sleep Medicine, University of Kansas Medical Center, Kansas City, KS, USA; m Baylor College of Medicine, Houston, TX, USA; n Guy's Severe Asthma Centre, School of Immunology & Microbial Sciences, Guy's Hospital, King's College London, London, UK; o AstraZeneca, Cambridge, UK; p GlaxoSmithKline, London, UK; q Sanofi, Cambridge, MA, USA; r Regeneron Pharmaceuticals, Tarrytown, NY, USA; s AbbVie, North Chicago, IL, USA; t Genentech, South San Francisco, CA, USA; u Novartis Healthcare Private, Hyderabad, India; v Medical Research Institute of New Zealand, Wellington, New Zealand; w Julius Center for Health Sciences and Primary Care, University Medical Center Utrecht, Utrecht, Netherlands

## Abstract

**Background:**

Asthma symptoms often guide disease assessment and management, but their prognostic and predictive value is unclear. We evaluated the extent to which symptom burden measured by the 5-item Asthma Control Questionnaire (ACQ-5) predicts future severe asthma attacks and response to anti-inflammatory therapy.

**Methods:**

We conducted an individual participant data meta-analysis of selected randomised controlled trials and translational observational cohort studies of asthma of varying severity. Primary analyses used the ORACLE2 patient-level meta-analysis (n=6513) of control-group participants from 22 randomised controlled trials. Additional datasets from studies assessing type 2 targeting anti-inflammatory therapies were included on the basis of availability of data, to provide insight into the association between ACQ-5 and sputum cell counts or mediator data. Additional datasets were the DREAM intravenous mepolizumab group (n=461); a cross-sectional severe asthma cohort (SA-OT, n=74); and two acute asthma cohorts (PRISMA, biologic-naive, n=53; BOOST, anti-interleukin-5-treated, n=60). Associations between baseline ACQ-5 scores and clinical, physiological, and inflammatory profiles, asthma attack risk, and anti-inflammatory treatment responses were examined.

**Findings:**

Across five datasets encompassing 7161 distinct participants, asthma severity, lung function, inflammatory profiles, comorbidities, and ACQ-5 results varied widely. The proportion of patients with high symptom burden (ACQ-5 score >1·5) ranged from 39% to 100%. Baseline ACQ-5 showed no consistent cross-sectional association with other clinical, physiological, or inflammatory asthma features. Each 0·5-point increase in baseline ACQ-5 score was associated with a modest increase in future asthma attack risk (adjusted rate ratio [aRR] 1·09 [95% CI 1·06–1·12]; Δ *R*^2^=0·02 *vs* multivariable prediction model without ACQ-5). Baseline ACQ-5 score did not alter relative and absolute attack risk reduction from intravenous mepolizumab in DREAM. By contrast, patients with high blood eosinophil counts and high fractional exhaled nitric oxide (FeNO) had the highest relative and absolute risk reduction (2·81 *vs* 1·17 attacks; aRR 0·38 [95% CI 0·25–0·57]). In the PRISMA and BOOST datasets, ACQ-5 was not associated with post-corticosteroid lung function change. Across studies, relative and absolute treatment effects showed consistent associations with blood eosinophil counts and FeNO.

**Interpretation:**

In a large, individual patient-level meta-analysis of randomised controlled trials and translational prospective observational cohort studies, we observed little alignment of symptom burden with other clinical, physiological, or biological features of asthma and modest prognostic value for severe attacks. Conversely, type 2 biomarkers more reliably identified patients at high risk and those likely to respond to treatment. Symptoms might require contextual interpretation to guide anti-inflammatory escalation in asthma.

**Funding:**

National Institute for Health and Care Research, Association Pulmonaire du Québec, Fonds de Recherche du Québec—Santé, and The Academy of Medical Sciences.

## Introduction

Asthma affects approximately 300 million people worldwide and is a major cause of morbidity, mortality, and health-care costs.^1^ The goal of asthma management should be the best possible outcomes for the individual, while minimising treatment-related toxic effects, including those associated with high-dose inhaled corticosteroids^2^, ^3^, ^4^ and oral corticosteroids.^4^ Although multimodal assessment is recommended,^1^, ^5^ symptom-only approaches are frequently used to stratify the risk of asthma attacks and escalate or decrease anti-inflammatory therapy.

Generally, symptoms are central to disease diagnosis and monitoring, yet they might not reliably serve as indicators for necessary secondary prevention in a wide range of common chronic diseases beyond asthma. Asymptomatic individuals with established disease might remain at high risk of adverse outcomes and require more preventive therapy. For example, high blood pressure is associated with preventable cerebrovascular events, and treatment of hyperglycaemia can help to avoid complications of diabetes. Conversely, symptoms might be disproportionate to disease pathology when factors such as obesity, deconditioning, breathing pattern disorders, or anxiety contribute to respiratory symptom burden, potentially leading to an increase in treatment exposure without meaningful clinical benefit. Accordingly, the management of major chronic disorders relies on multimodal evaluation.^6^, ^7^, ^8^

**Research in context**

**Evidence before this study**

We searched PubMed from database inception to Feb 2, 2026, for clinical trials, systematic reviews, and meta-analyses assessing asthma attacks, with the terms “asthma”, “exacerbation”, “risk”, and “factors”, restricting to English and French reports. Of 204 publications identified, ten reported multivariable prediction models. Five studies quantified the prognostic value of symptom scores, four reported statistically significant associations, and none assessed differential treatment effects associated with symptom burden. Most used the Asthma Control Questionnaire (ACQ). Additionally, we reviewed a 2025 annual independent international expert (Global Initiative for Asthma) literature review that highlighted the prognostic value of symptoms for predicting asthma attacks. Based on four key publications, symptom monitoring was recommended as part of multimodal assessment to guide treatment escalation. Two of these publications were restricted to severe asthma populations, whereas the two others analysed broader populations using more rigorous methods; the broader and more rigorous studies reported modest prognostic effects. No previous study simultaneously examined cross-sectional associations with underlying type-2 biomarkers and asthma parameters, incremental prognostic value, and differential treatment response across chronic and acute settings. In summary, although ACQ-measured symptoms generally showed prognostic value for asthma attacks, their ability to identify treatment-responsive pathology and patients—both on relative and absolute scales—remains unclear.

**Added value of this study**

We analysed data from multiple randomised controlled trials and translational observational cohort studies across diverse clinical settings, including 7161 distinct patients. First, in a multi-study, cross-sectional analysis, baseline symptom burden was not associated with other measures of clinical severity, lung function, or biological disease activity. Second, among patients with chronic asthma randomly assigned to control groups in 22 randomised controlled trials included in the ORACLE2 individual patient-level meta-analysis, multivariable models with or without symptom burden had negligible differences in explained variability in asthma attack risk. Finally, in a post-hoc analysis of a placebo-controlled trial of intravenous mepolizumab in patients with severe eosinophilic asthma, symptom burden alone did not add value in terms of identifying patients who are more treatment-responsive. Similar findings were observed in prospective observational studies of severe asthma attacks assessed before and after treatment with oral corticosteroids, both in biologic-naive and biologic-treated patients. Across studies, type-2 inflammatory biomarkers, specifically blood eosinophil count and fractional exhaled nitric oxide, showed strong and graded performance and identified patients at high risk and with greater relative and absolute therapeutic responses. To our knowledge, this study is the largest to evaluate the mechanistic, prognostic, and theragnostic value of symptom burden in asthma, highlighting particular limitations of symptom-only assessment.

**Implications of all the available evidence**

The available evidence suggests that asthma symptoms might not reflect other underlying clinical, physiological, and biological features of the disease. Although symptom burden is associated with an increased risk of asthma attacks, our findings highlight the need for individual assessment of contributing clinical and pathophysiological factors to tailor preventive interventions. Accordingly, our study supports the development of a multivariable clinical prediction model for asthma attacks without explicit symptom-based stratification, analogous to approaches used in cardiovascular disease and other chronic illnesses. Decisions regarding therapeutic escalation in both stable asthma and acute asthma attacks could benefit from greater emphasis on type-2 inflammatory biomarkers. These findings support a move towards more individualised clinical decision making in asthma, in which symptoms are considered in context rather than used as the sole determinant of management.

In patients with asthma, the dissociation between symptoms and objective markers of disease severity has prompted calls to redefine airway disease by prioritising underlying pathological features.^5^ Nevertheless, symptom assessment remains central to asthma management, with tools such as the Asthma Control Questionnaire (ACQ) used for longitudinal monitoring; a 0·5-point change in ACQ score reflects a clinically meaningful difference in symptom burden.^9^ Uncontrolled symptoms continue to be an inclusion criterion for most clinical trials on asthma,^10^, ^11^, ^12^, ^13^, ^14^, ^15^, ^16^, ^17^, ^18^, ^19^, ^20^, ^21^, ^22^, ^23^ and symptom deterioration lasting more than 48 h despite increased reliever use remains a recommended trigger for oral corticosteroid initiation, with or without objective airflow assessment.^1^ Whether these recommendations align with underlying inflammatory biology and therapeutic responsiveness, however, is uncertain. This uncertainty is compounded by the potential for poorly targeted treatment and substantial toxic effects from corticosteroids, including pneumonia, osteoporosis, diabetes, cataracts, and adrenal insufficiency.^2^, ^3^, ^4^, ^24^, ^25^

By contrast, type 2 inflammation is a key immunopathological pathway in many patients with asthma, reflected by elevated blood eosinophil counts, or high fractional exhaled nitric oxide (FeNO), or both. These type 2 biomarkers are robust indicators of airway pathology,^26^, ^27^ future risk of asthma attacks,^28^ and lung function decline,^29^, ^30^ and are predictive of a patient's response to anti-inflammatory therapies.^31^, ^32^, ^33^

To better understand the strengths and limitations of symptom-based and biomarker-based assessment, we aimed to investigate their relationship with asthma attack rates and outcomes of anti-inflammatory treatment escalation in a diverse population of patients with asthma.

## Methods

### Study objectives and design

In this individual patient-level meta-analysis, we aimed to evaluate the cross-sectional, prognostic, and predictive value of the 5-item ACQ (ACQ-5)^9^ across randomised controlled trials (RCTs) and translational observational cohort studies.

Our specific objectives were, first, to examine cross-sectional associations between ACQ-5 scores and other clinical, physiological, and inflammatory markers of asthma activity; second, to quantify the association between baseline ACQ-5 scores and subsequent risk of severe asthma attacks, and quantify the incremental prognostic value of the ACQ-5 in multivariable modelling; and third, to assess whether ACQ-5 scores indicate responsiveness to anti-inflammatory treatment in both relative and absolute terms.

We primarily used datasets generated from ORACLE2, a previous systematic review and patient-level meta-analysis of the control groups of randomised controlled trials (RCTs) in asthma conducted from 1993 to 2021.^28^, ^34^ Additional datasets from studies assessing anti-inflammatory therapies targeting type 2 inflammation were included on the basis of data availability rather than through a formal systematic review. Permission was granted to analyse one intervention group of a trial included in ORACLE2 (DREAM^35^). Three other studies available for analysis (via authors SC and IDP as guarantors of these studies) provided insight into the association between ACQ-5 scores and sputum cell counts or mediator data.^26^, ^32^, ^33^, ^36^, ^37^ Despite academic investigator requests, no other intervention group data for trials included in the ORACLE2 meta-analysis were available. No interventional studies for which the main authors had permission to analyse the data were excluded.

### Study populations

We used datasets from the ORACLE 2 individual patient data meta-analysis^28^, ^34^ and the DREAM,^35^ Severe Asthma on Optimal Therapy (SA-OT),^26^ PRISMA,^33^ and BOOST^32^ studies. Primary analyses used data from the ORACLE2 patient-level meta-analysis (PROSPERO CRD42021245337)^28^, ^34^ of only control-group participants aged 12 years or older from 22 RCTs^10^, ^11^, ^12^, ^13^, ^14^, ^15^, ^16^, ^17^, ^18^, ^19^, ^20^, ^21^, ^22^, ^23^, ^35^, ^38^, ^39^, ^40^, ^41^ with at least 6 months of follow-up. Control groups were analysed as they represented the patients with the lowest anti-inflammatory intensity after random assignment (ie, those without use of inhaled corticosteroids, lowest-dose inhaled corticosteroids, or inhaled corticosteroids plus placebo). Data were extracted by SC and FLM, with extraction programming code previously made available.^28^ Three analytical populations (ie, subgroups) were defined from the ORACLE2 patients: the full ORACLE2 subgroup (n=6513); the any-ACQ subgroup (n=750), which included participant data from five trials without symptom-based inclusion criteria;^35^, ^38^, ^39^, ^40^, ^41^ and the ACQ-items subgroup (n=3858), which included participant data from 14 trials providing data for individual ACQ-5 item responses.^11^, ^12^, ^18^, ^19^, ^20^, ^22^, ^23^, ^35^, ^38^, ^39^, ^40^, ^41^

DREAM^35^ enrolled adolescents and adults with severe eosinophilic asthma and recurrent attacks who had evidence of an underlying eosinophilic phenotype but no requirement for a symptom or ACQ threshold. 621 participants were randomly assigned 1:3 to placebo or intravenous mepolizumab (anti-interleukin [IL]-5) every 4 weeks for 52 weeks. All mepolizumab groups represented unlicensed doses pooled for analysis, as done previously.^42^ Data were extracted by GlaxoSmithKline by use of the same programming code as for the ORACLE2 data transfer,^28^ with the additional transfer of patient-level data from the interventional group for the current analysis.

SA-OT is a previously analysed^26^ pooled cross-sectional study of 74 patients with optimised severe asthma. This study contributed detailed clinical and biological data from patients with verified adherence to high-intensity inhaled corticosteroids, systemic corticosteroids, or both, regardless of their residual symptom burden. Assessments followed treatment optimisation to suppress FeNO either by use of a structured protocol^43^ or after participation in the UK Refractory Asthma Stratification programme (RASP-UK) trial.^44^, ^45^ Data were extracted by SC and PB.

PRISMA^33^, ^37^ was a prospective, observational cohort study of 53 biologic-naive patients with severe asthma attacks, enriched for airway inflammatory phenotyping. Inclusion required objectively diagnosed asthma and an asthma attack requiring oral corticosteroids based on Global Initiative for Asthma (GINA) criteria after at least 48 h of persistent symptoms.^1^ Assessments were conducted before and after administration of oral corticosteroids, with post-bronchodilator FEV_1_ (L) as the primary outcome. Data were extracted by SL-P and CC-P.

BOOST^32^, ^36^ was a similar prospective cohort enrolling 60 patients who had a severe asthma attack while receiving anti-IL-5 or anti-IL-5-receptor biologics, defined by persistent symptoms similarly to PRISMA. Visits before and after oral corticosteroid administration (day 0 and day 7) were analysed, with post-bronchodilator FEV_1_ change herein assigned as the primary outcome. Data were extracted by IH.

Ethics approval and written informed consent were obtained for all studies.^26^, ^28^, ^32^, ^33^, ^35^ The present study used de-identified data for secondary analysis. Additional ethics approval for the ORACLE2 meta-analysis was deemed unnecessary by the Oxford Tropical Research Ethics Committee.

### Outcomes, variables, and specimens

In the chronic asthma studies, ORACLE2 and DREAM, the primary outcome was severe asthma attacks, defined as episodes requiring at least 3 days of systemic corticosteroids.^46^ In asthma attack studies, PRISMA and BOOST,^32^, ^33^ the primary outcome was post-bronchodilator FEV_1_ change (in litres) at day 7 post-oral corticosteroids.

Baseline variables were selected on the basis of statistical significance in previous modelling of the risk of severe asthma attacks in the ORACLE2 dataset.^28^ The patient-reported measurement of interest, baseline ACQ-5 score, assesses five symptom domains on a 7-point scale, with mean scores of 1·5 or higher indicating high symptom burden and 0·5 points representing the minimal clinically important difference.^9^, ^47^ Other variables we assessed were sex assigned at birth, BMI, presence of severe asthma attack in the previous 12 months, GINA treatment step (ordinal 1–5), FEV_1_ (% predicted), FEV_1_/forced vital capacity ratio (FVC) ratio, blood eosinophil count, FeNO, smoking history, allergic rhinitis, chronic rhinosinusitis without nasal polyps, and chronic rhinosinusitis with nasal polyps. The GINA treatment step was defined by prescribed treatment intensity (step 1: mild asthma with no controller to step 5: severe asthma or high-intensity regimen; appendix p 5. Comorbidities were patient-reported.

Sputum induction and differential counts were collected in the SA-OT, PRISMA, and BOOST datasets by use of standardised protocols.^26^, ^36^, ^37^, ^43^, ^44^ In sputum supernatant for the SA-OT and PRISMA datasets, inflammatory proteins were measured in duplicates with multiplex electrochemiluminescent assays (Meso Scale Discovery, Rockville, MD, USA) or single enzyme-linked immunosorbent assays (Cayman-Chemical, Ann Arbor, MI, USA); prostaglandin-D2-only). Risk of bias and heterogeneity was not assessed in the translational observational cohort studies.

### Statistical analysis

Data extraction for ORACLE2 and DREAM followed those previously reported.^28^ Duplicate inclusion of data should not be possible because the same programming code was executed independently by GlaxoSmithKline for the ORACLE2 placebo group analyses. For the present analysis, GlaxoSmithKline additionally provided patient-level data from the interventional group, which were not included in the original ORACLE2 analyses. Missingness was summarised per dataset. ORACLE2 used multiple imputation by chained equations (ten datasets) with Rubin's rules applied. Other datasets were analysed with complete cases, as per the original study reports.^26^, ^32^, ^33^, ^35^ Eosinophil count, FeNO, and other inflammatory mediators were log-transformed (replacing null values by 0·5 × lower limit of detection).

In terms of participant characterisation, categorical variables were summarised as frequencies with percentages and continuous variables as median with IQR. ACQ-5 score distributions were explored with density plots.

To assess the cross-sectional associations between ACQ-5 scores and underlying asthma characteristics, cross-sectional regression analyses were conducted in the ORACLE2 any-ACQ dataset (ie, the subset of control group participants of trials not selecting according to ACQ-5) and the SA-OT, PRISMA, and BOOST datasets, with baseline ACQ-5 score as the dependent variable and other baseline clinical, physiological, and biological measures as independent variables. Study-specific regression estimates were pooled in a two-stage random-effects meta-analysis.

To assess the prognostic value of ACQ-5 scores to predict asthma attacks, in the full ORACLE2 and any-ACQ datasets, we used univariable and multivariable negative binomial models with log(time) offset to estimate associations between baseline ACQ-5 scores and attack risk. We used restricted cubic splines with three knots to visualise associations. Rate ratios (RRs) with 95% CIs were reported per 0·5-point ACQ-5 increase (minimal clinically important difference).^9^ Likewise, we derived ACQ-5 values corresponding to 20% and 40% asthma attack rate increases.^48^

Prognostic performance was evaluated in mixed-effect models (trial set as random intercept) using changes in Nagelkerke *R*^2^ when excluding ACQ-5, attack history, or biomarkers, and by comparing models using each prognostic factor alone.

In the ORACLE2 ACQ-items dataset, univariable and adjusted associations for each ACQ-5 item and all pairwise interactions were tested.

To assess the theragnostic value of ACQ-5 scores to predict treatment effects, in the DREAM dataset, treatment effects on annualised attack rates were analysed with multivariable negative binomial models. Datasets were defined by quartiles for ACQ-5 score, blood eosinophil count, and FeNO (<Q1, Q1–Q3, and >Q3), and blood eosinophil–FeNO composite type 2 (T2) ordinal strata^33^, ^49^ (T2-low–low, ie, <0·15 × 10^9^ cells/L and <25 parts per billion (ppb); T2-high–high, ie, ≥0·30 × 10^9^ cells/L and ≥35 ppb; or T2-mid, ie, not meeting low–low or high–high criteria).

In the PRISMA and BOOST datasets, changes in post-bronchodilator FEV_1_ were assessed for associations with baseline (pre-attack) variables with multivariable linear regression models. Similar subgroup analyses were conducted.

In terms of adjustment strategies, full multivariable adjustment was applied in ORACLE2 and DREAM with 12 baseline prognostic factors, which were significant in the ORACLE2 dataset,^28^ with additional adjustment for the participant's source trial in ORACLE2. The SA-OT, PRISMA, and BOOST regression models were adjusted for baseline FEV_1_, ACQ-5 score, blood eosinophil count, and FeNO. When T2 ordinal status was evaluated as a covariate, blood eosinophils and FeNO were removed.

In terms of sample size considerations, no formal sample size was calculated. Considering 12 covariates in the ORACLE2 and DREAM datasets, event-per-variable (EPV) ratios were 384 in the full ORACLE2 dataset, 35 in the any-ACQ dataset, 204 in the ACQ-items dataset, and 24 or higher in DREAM, exceeding accepted thresholds (EPV >10). Simpler models were used in smaller datasets: four covariates were used in the SA-OT, PRISMA, and BOOST datasets.

Regarding significance and software, all analyses calculated 95% CI. False discovery rate correction was applied to the ACQ-5 item interaction analyses (false discovery rate <0·05). Analyses were conducted in R version 4.4.2. Prognostic modelling across the multiply imputed ORACLE2 dataset used code generated with Claude (Sonnet 5), which was reviewed and approved by a human author (SC). All other code was written by human authors (SL-P, PC, and SC). Code is available on GitHub.

### Role of the funding source

The funders of the study had no role in study design, data collection, data analysis, data interpretation, or writing of the report.

## Results

Of the parent-study populations, we analysed all 6513 ORACLE2 participants (who contributed 5482 total follow-up years);^28^ 619 of 621 DREAM participants^35^ (including 158 placebo participants who were also part of ORACLE2 and excluding two patients who did not share data), all 74 SA-OT participants,^26^ all 53 PRISMA participants,^33^ and all 60 BOOST participants,^32^ for a total of 7161 distinct participants. Missing data analysis, imputation results in ORACLE2, and the underlying studies, risk of bias, and heterogeneity of the underlying studies in ORACLE2 have been reported previously.^28^

Baseline participant characteristics are shown in the table, and study characteristics are provided in the appendix (pp 6–11). Across studies, participants had heterogeneity in asthma severity, lung function, type 2 inflammatory biomarkers, GINA treatment step, and comorbidity profiles. Of the 22 RCTs included in the ORACLE2 dataset, only five^35^, ^38^, ^39^, ^40^, ^41^ enrolled patients irrespective of baseline symptom burden. The proportion of patients with ACQ-5 scores higher than 1·5 (indicating high symptom burden) varied widely, ranging from 100% in the BOOST participants to 39% in the ORACLE2 any-ACQ participants subgroup. Additional characteristics are shown in the appendix (pp 12–15).

**Table:** Baseline characteristics

		ORACLE2*		**DREAM**		**SA-OT (N=74)**	**PRISMA (N=53)**	**BOOST (N=60)**	**p value**
		Full (N=6513)	Any ACQ (N=750)	Placebo (N=158)	Mepolizumab (N=461)				
Sex		..	..	..	..	..	..	..	0·0005
	Female	4140 (64%)	422 (56%)	98 (62%)	290 (63%)	33 (45%)	34 (64%)	38 (63%)	..
	Male	2370 (36%)	328 (44%)	60 (38%)	171 (37%)	41 (55%)	19 (36%)	22 (37%)	..
	Missing	3	0	0	0	0	0	0	..
Age, years		..	..	..	..	..	..	..	<0·0001
	Median	50 (39–59)	42 (27–55)	47 (39–55)	50 (43–58)	55 (46–63)	49 (41–60)	56 (48–64)	..
	Missing	0	0	3	0	0	0	0	..
Race		..	..	..	..	..	..	..	<0·0001
	White	3825 (59%)	634 (85%)	142 (90%)	414 (90%)	65 (88%)	50 (94%)	51 (85%)	..
	Asian	502 (8%)	45 (6%)	8 (5%)	26 (6%)	3 (4%)	1 (2%)	7 (12%)	..
	Black or African American	267 (4%)	10 (1%)	7 (4%)	18 (4%)	2 (3%)	0	1 (2%)	..
	Other or multiple	187 (3%)	61 (8%)	1 (1%)	3 (1%)	4 (5%)	2 (4%)	1 (2%)	..
	Missing	1732	0	0	0	0	0	0	..
BMI, kg/m^2^		..	..	..	..	..	..	..	<0·0001
	Median	28 (25–31)	27 (24–30)	27 (24–31)	28 (24–32)	28 (25–32)	28 (25–34)	28 (24–33)	..
	Missing	2739	0	1	0	0	0	0	..
Mean ACQ-5 score		..	..	..	..	..	..	..	<0·0001
	Median	2·4 (1·8–3·0)	1·2 (0·6–2·0)	2·6 (1·8–3·2)	2·4 (1·4–3·2)	1·6 (0·6–2·2)	3·6 (2·6–4·2)	3·6 (3·0–4·4)	..
	<1·5	929 (14%)	443 (59%)	29 (19%)	117 (26%)	35 (47%)	3 (6%)	0	..
	Missing	458	20	5	9	0	0	0	..
Severe attack in past year		..	..	..	..	..	..	..	<0·0001
	Yes	5210 (80%)	266 (36%)	157 (100%)	461 (100%)	48 (65%)	40 (75%)	39 (65%)	..
	Missing	0	0	1	0	0	0	0	..
Treatment step		..	..	..	..	..	..	..	<0·0001
	1	226 (3%)	226 (30%)	0	0	0	1 (2%)	0	..
	2	315 (5%)	315 (42%)	0	0	0	3 (6%)	0	..
	3	859 (13%)	0	0	0	0	6 (11%)	0	..
	4	2555 (39%)	0	0	0	0	15 (28%)	0	..
	5	2558 (39%)	209 (28%)	158 (100%)	461 (100%)	0	28 (53%)	60 (100%)	..
	Missing	0	0	0	0	74	0	0	..
FEV_1_ prebronchodilator, % predicted		..	..	..	..	..	..	..	<0·0001
	Median	64·0 (53·0–73·9)	66·5 (52·8–80·0)	61·4 (47·4–69·7)	60·5 (49·1–71·6)	82·8 (75·7–97·6)	79·7 (66·0–88·3)	69·5 (53·3–85·3)	..
	Missing	565	525	3	0	0	0	0	..
FEV_1_/FVC ratio prebronchodilator, %		..	..	..	..	..	..	..	<0·0001
	Median	64·2 (56·0–72·3)	65·9 (55·8–75·0)	61·4 (54·1–70·6)	63·7 (55·0–72·3)	70·3 (61·4–79·2)	71·1 (66·6–75·3)	66 (54·8–76·0)	..
	Missing	710	525	3	0	0	0	0	..
Blood eosinophil count, × 10^9^/L		..	..	..	..	..	..	..	<0·0001
	Median	0·25 (0·14–0·42)	0·25 (0·15–0·39)	0·34 (0·16–0·61)	0·27 (0·15–0·49)	0·25 (0·13–0·50)	0·21 (0·10–0·36)	0·02 (0–0·06)	..
	Missing	80	5	3	0	0	0	0	..
FeNO, ppb		..	..	..	..	..	..	..	<0·0001
	Median	23 (14–42)	30 (18–60)	33 (19–59)	30 (18–54)	42 (27–63)	18 (11–37)	48 (18–80)	..
	Missing	532	5	7	6	0	0	0	..
Smoking status		..	..	..	..	..	..	..	<0·0001
	Never	5197 (80%)	540 (72%)	123 (78%)	362 (79%)	54 (73%)	41 (77%)	43 (72%)	..
	Ex	1161 (18%)	174 (23%)	34 (22%)	99 (21%)	17 (23%)	12 (23%)	17 (28%)	..
	Current	38 (1%)	36 (5%)	0	0	3 (4%)	0	0	..
	Missing	117	0	1	0	0	0	0	..
Allergic rhinitis†		..	..	..	..	..	..	..	<0·0001
	Yes	2501 (48%)	10 (62%)	0	0	0	33 (62%)	8 (13%)	..
	No	2671 (52%)	6 (38%)						
	Missing	1341	734‡	158	461	74	0	0	..
CRSsNP†		..	..	..	..	..	..	..	<0·0001
	Yes	441 (11%)	47 (22%)	29 (18%)	67 (15%)	0	15 (28%)	1 (2%)	..
	No	3711 (89%)	165 (78%)						
	Missing	2361	538	0	0	74	0	0	..
CRSwNP†		..	..	..	..	..	..	..	<0·0001
	Yes	343 (8%)	20 (9%)	17 (11%)	41 (9%)	0	11 (21%)	14 (23%)	..
	No	3825 (92%)	194 (91%)						
	Missing	2345	536	0	0	74	0	0	..

Data are n (%) or median (IQR) unless otherwise stated. p values test differences between all datasets and are from ANOVA for continuous variables or Fisher's exact tests for categorical data, except for blood eosinophil count and FeNO, which are Kruskal–Wallis tests. Baseline values are pre-random assignment (stable-state) for ORACLE2, CAPTAIN, and DREAM; single-visit value for SA-OT; and attack day 0 (pre-corticosteroid burst) for PRISMA and BOOST. DREAM placebo patients are also represented in the ORACLE2 full dataset column. Baseline characteristics of the ORACLE2 items dataset are presented in the appendix (p 13). ACQ=Asthma Control Quesitonnaire. ACQ-5=5-item Asthma Control Questionnaire. CRSsNP=chronic rhinosinusitis without nasal polyps. CRSwNP=chronic rhinosinusitis with nasal polyps. FeNO=fractional exhaled nitric oxide. FF=fluticasone furoate. FVC=forced vital capacity. ppb=parts per billion. SA-OT=Severe Asthma on Optimal Therapy.

* Percentages are not provided for missing data as values have been imputed.

† Patient-reported.

‡ This estimate is based on imputed data and reported for completeness, but little importance should be attached to it due to the high amount of missing data

The association between baseline ACQ-5 scores and other baseline participant characteristics across studies that did not require a minimal ACQ score for participant inclusion is shown in figure 1 and the appendix (pp 16–19). In the pooled random-effects meta-analysis, symptom burden was associated with younger age (adjusted mean difference [aMD] per 10 years older age –0·12 [95% CI –0·17 to –0·08]), female sex (0·28 [0·04 to 0·53]), and smoking history (ordinally from never smoked to ex-smoking to current smoking 0·17 [0·06 to 0·28]). We found no association between symptoms and inflammatory mediators (ie, blood eosinophils, FeNO, and sputum eosinophils or cytokines); this dissociation was consistent across chronic and acute datasets.

**Figure 1:**
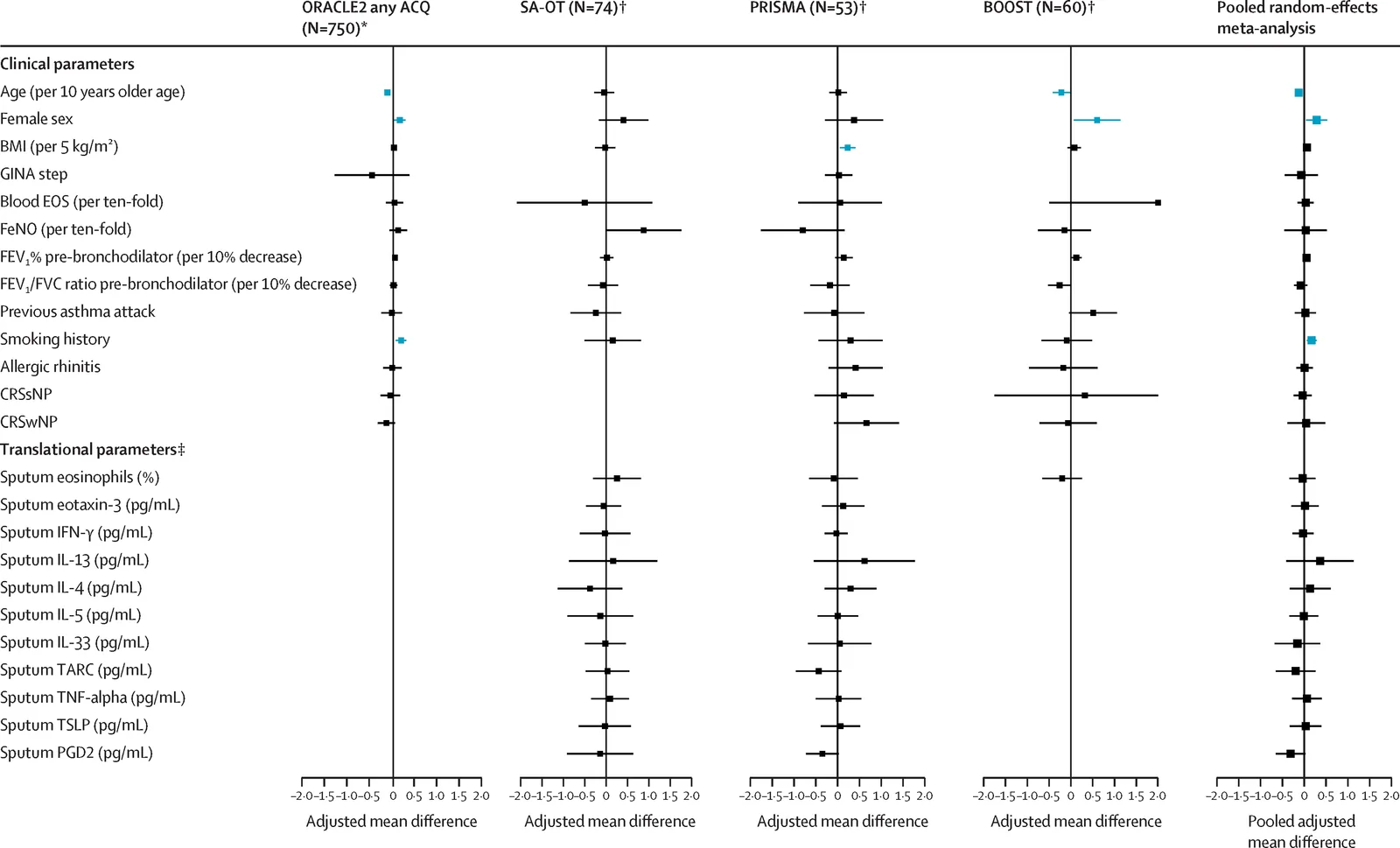
Cross-sectional association between mean differences in ACQ-5 scores and other clinical and translational characteristics Statistically significant mean differences are shown in blue. A table with estimates, 95% CI, and numbers analysed and missing is provided in the appendix (pp 14–15). ACQ-5=5-item Asthma Control Questionnaire. PreBD=prebronchodilator. CRSsNP=chronic rhinosinusitis without nasal polyposis. CRSwNP=chronic rhinosinusitis with nasal polyposis. EOS=eosinophil count. FeNO=fractional exhaled nitric oxide. FVC=forced vital capacity. GINA=Global Initiative for Asthma. IL=interleukin. PGD2=prostaglandin D2. preBD=prebronchodilator. TARC=thymus and activation-regulated chemokine. TSLP=thymic stromal lymphopoietin. *Mean differences are adjusted for sex, BMI, occurrence of severe attack in preceding 12 months, GINA treatment step, FEV_1_%, FEV_1_/FVC ratio, blood eosinophil count, FeNO, smoking history, allergic rhinitis, CRSsNP, CRSwNP, and enrolled trial. †Mean differences are adjusted for blood eosinophil count, FeNO, and FEV_1_ percentage. ‡All translational parameters are per 10-fold increase.

In terms of prognostic modelling, the full ORACLE2 dataset, each 0·5-unit increment in baseline ACQ-5 score was associated with an increase in risk of severe asthma attack (adjusted rate ratio [aRR] 1·09 [95% CI 1·06–1·12]; figure 2; appendix p 20). A standardised change of the magnitude of the IQR (1·8–3·0, a 1·2-point difference) was associated with an aRR of 1·23 (95% CI 1·16–1·31). Clinically meaningful 20% and 40% asthma attack rate increases^48^ corresponded to increases in adjusted ACQ-5 scores of 1·06 (0·81–1·50) and 1·95 (1·50–2·77) units, respectively (appendix p 21). Additional estimates for ACQ-5 prognostic effects according to original categorisation of mean scores are provided in the appendix (p 22). Similar prognostic effects were observed when analysing the ORACLE2 any-ACQ dataset. In a complementary analysis in the ORACLE2 ACQ-items dataset, items showing the strongest univariable associations were wheezing (per 0·5-unit increment, 1·05 [1·02–1·09]) and dyspnoea (1·05 [1·00–1·09]), but these findings were not statistically significant after multivariable adjustment (1·03 [1·00–1·06] and 1·03 [0·99–1·08], respectively; appendix p 2).

**Figure 2:**
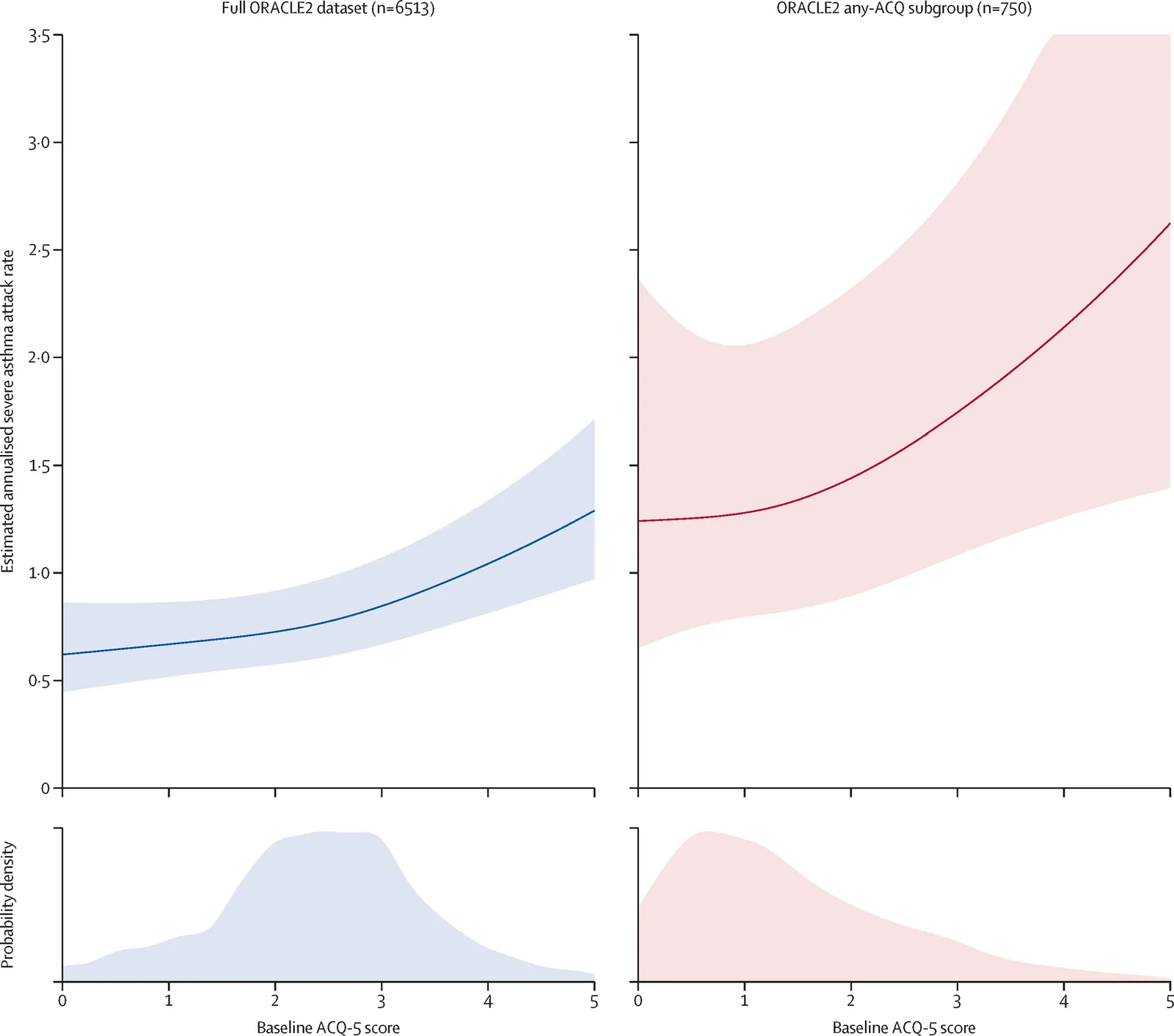
Association between baseline ACQ-5 and annualised severe asthma attack rate in the full ORACLE2 and ORACLE any-ACQ cohorts, with their associated density plots Models are adjusted for sex, BMI, occurrence of severe asthma attack in preceding 12 months, Global Initiative for Asthma treatment step, FEV_1_%, FEV_1_/FVC ratio, blood eosinophil count, FeNO, blood eosinophil count × FeNO, smoking history, allergic rhinitis, chronic rhinosinusitis with and without nasal polyposis, and enrolled trial. Results pooled across 10 multiply imputed datasets using Rubin's rules. ACQ-5=5-item Asthma Control Questionnaire. FeNO=fractional exhaled nitric oxide. FVC=forced vital capacity.

The full multivariable model explained 44% of the variance in asthma attacks (*R*^2^=0·44), whereas models based on single prognostic factors showed substantially lower explained variance: ACQ-only, *R*^2^ 0·18; attack history-only, *R*^2^ 0·21 (strongest single prognostic effect); biomarkers-only, *R*^2^ 0·18 (appendix p 24). Removing ACQ-5 resulted in only a small reduction in explained variance (Δ*R*^2^=0·02). By comparison, Δ*R*^2^ was 0·06 when attack history was excluded and 0·04 when type 2 biomarkers were excluded. Additional performance parameters are provided in the appendix (p 23).

Associations between baseline ACQ-5 and anti-inflammatory treatment effects in participants with stable severe eosinophilic asthma in DREAM are shown in figure 3. Overall, intravenous mepolizumab reduced severe asthma attacks compared with placebo (1·19 *vs* 2·11 attacks per year; aRR 0·55 [95% CI 0·44–0·70]). Relative and absolute treatment effects were similar across ACQ-5 quartiles, with no significant interaction when ACQ-5 was analysed continuously (p_interaction_=0·18). By contrast, treatment efficacy was strongly and consistently associated with high blood eosinophil count and high FeNO, assessed individually and in combination. In continuous interaction multivariable analyses, blood eosinophil count (p_interaction_=0·0010) and composite type 2 ordinal classification (p_interaction_=0·034) were significant, whereas FeNO was not (p_interaction_=0·056). No benefit of mepolizumab versus placebo was observed in the type 2 low–low stratum (aRR 1·11 [0·52–2·37]). Treatment benefit increased with higher biomarker measurements; patients with high blood eosinophil counts and high FeNO had the greatest relative and absolute risk reduction (2·81 *vs* 1·17 attacks per year; aRR 0·38 [0·25–0·57]).

**Figure 3:**
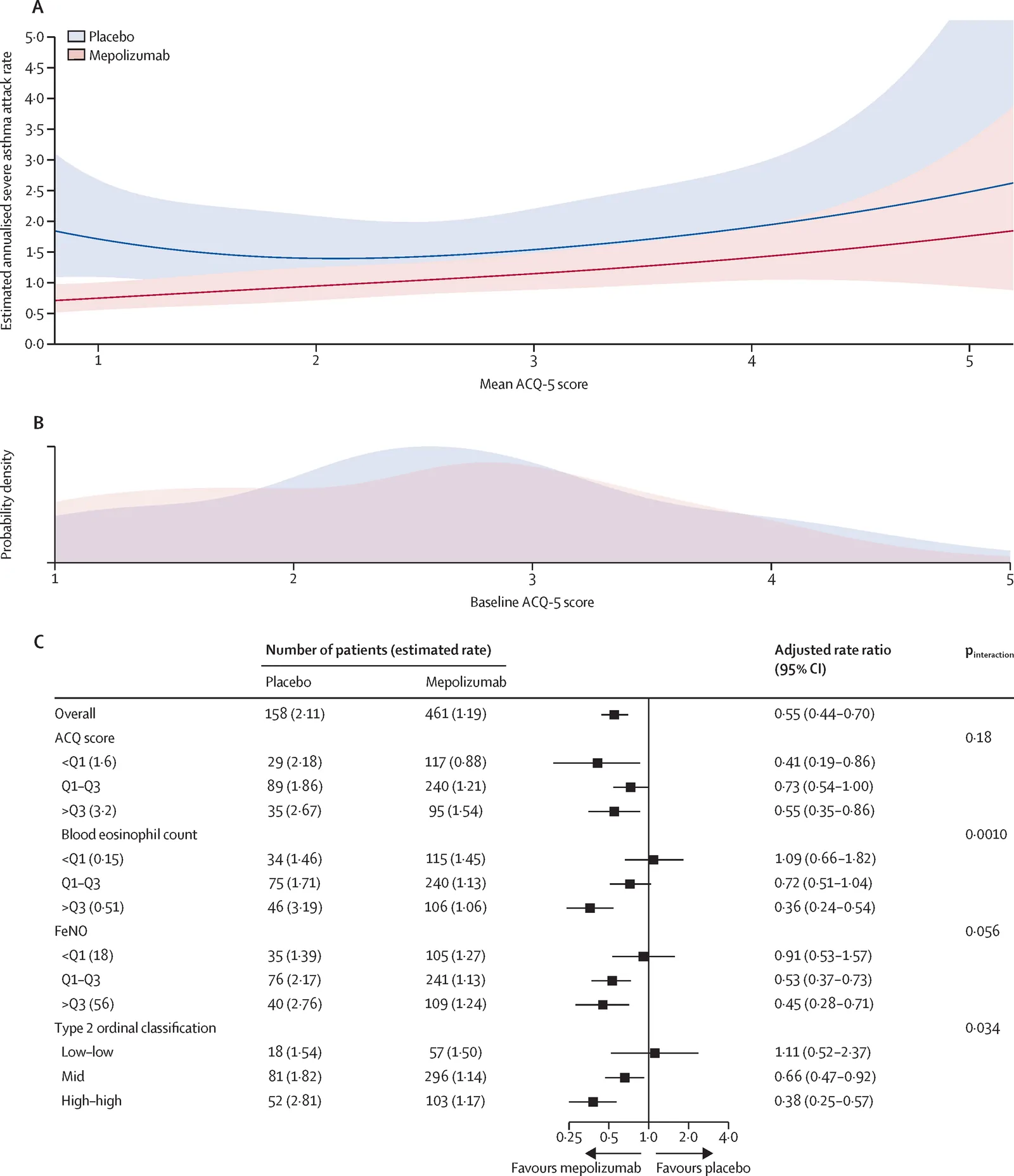
Anti-inflammatory treatment effects in the DREAM dataset (A) The restricted cubic spline for annualised severe asthma attack rates in intravenous mepolizumab versus placebo according to baseline ACQ-5 scores, adjusting for 12 covariables. (B) The distribution of baseline ACQ-5 as a density function. (C) Estimated attacks and adjusted treatment effects expressed as rate ratios, stratified by quartile (<Q1, Q1–Q3, >Q3 of the 461 participants) of baseline ACQ-5, blood eosinophil count, FeNO, and composite type 2 inflammatory ordinal classification. Estimates for T2 ordinal classification (T2-low–low, ie, blood eosinophil count <0·15 × 10^9^ cells/L and FeNO <25 ppb; T2-high–high, ie, ≥0·30 × 10^9^ cells/L and ≥35 ppb; or T2-mid, ie, not meeting low–low or high–high criteria) are from a model not adjusting for T2 status instead of blood eosinophil count and FeNO. p_interaction_ values are from multivariable models testing interaction with the continuous term of interest iteratively. ACQ-5=5-item Asthma Control Questionnaire. FeNO=fractional exhaled nitric oxide.

In PRISMA, post-oral corticosteroids (day 7) FEV_1_ changes in acute asthma were not associated with pre-oral corticosteroids ACQ-5 (per 0·5-unit increase, aMD 0·01 L [–0·02 to 0·05]; figure 4). Conversely, lung function responses showed stronger associations with the type 2 biomarkers blood eosinophils (per ten-fold increase, aMD 0·27 L [0·04 to 0·51]) and the composite type 2 ordinal stratification (per strata increase, aMD 0·18 L [0·07 to 0·29]). Expressed per IQR-equivalent increase for blood eosinophils (0·26  × 10^9^ cells per L), the associated adjusted mean difference in lung function was 0·10 L (0·02 to 0·19).

**Figure 4:**
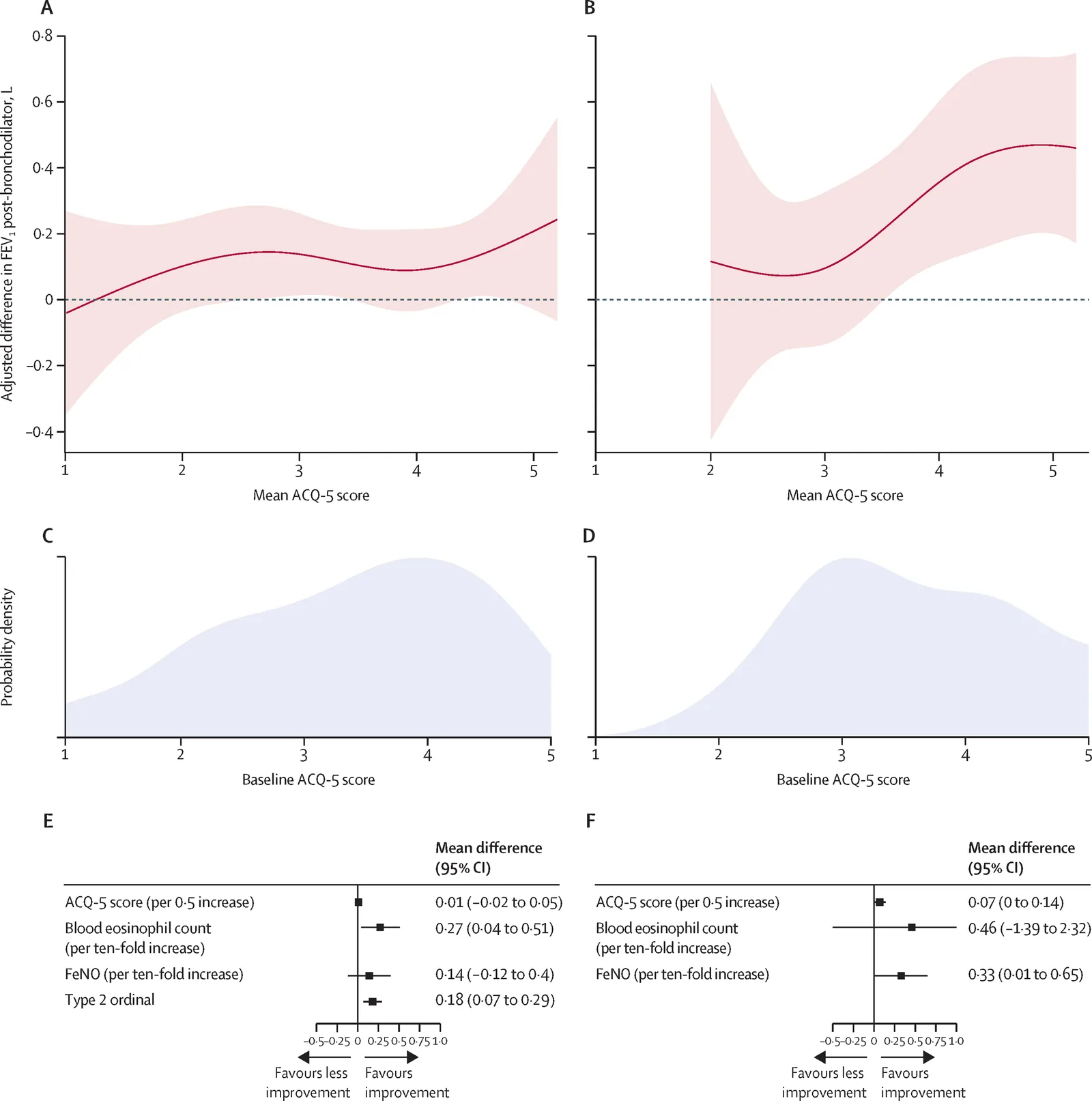
Oral corticosteroid treatment effects in asthma attacks Biologic-naive patients in PRISMA (A, C, and E) and patients treated with anti-IL-5 or anti-IL-5 receptor biologics in BOOST (B, D, and F), stratified according to attack (day 0) ACQ-5, blood eosinophil count, FeNO, and composite type 2 inflammatory ordinal classification. Analyses are adjusted for baseline (pre-attack) FEV_1_, ACQ-5 mean score and either blood eosinophil count plus exhaled nitric oxide or T2 ordinal classification. ACQ-5=5-item Asthma Control Questionnaire. FeNO=fractional exhaled nitric oxide.

In BOOST, attack (day 0) ACQ-5 showed a non-significant association with change in FEV_1_ post-oral corticosteroids (day 7). By contrast, a significant association was found between FeNO and FEV_1_ change (per ten-fold increase, aMD 0·33 L [95% CI 0·01–0·65]). Expressed per IQR-equivalent increase (63 ppb), the associated adjusted mean difference in lung function was 0·15 L (0·00–0·29).

Post-hoc theragnostic analyses per ACQ item for DREAM and PRISMA are provided in the appendix (pp 3–4). No per-item data were available for BOOST participants.

## Discussion

Across independent RCTs and translational observational cohort studies in asthma, symptom burden measured by the ACQ-5 was associated with modest incremental prognostic value for subsequent severe attacks. This association was found without consistent cross-sectional links between symptoms and other objective clinical, physiological, or biological features of asthma. In our analyses, symptom burden alone did not consistently identify patients who derived greater relative or absolute benefit from anti-inflammatory escalation, whether with a type 2 targeted biologic in chronic asthma or with a corticosteroid burst during an attack. By contrast, type 2 inflammatory biomarkers, specifically blood eosinophil counts and FeNO, showed strong and consistent prognostic and predictive performance in both relative and absolute terms. Although consistent, these observations were derived from post-hoc and dataset assessments, warrant cautious interpretation, and do not diminish the importance of assessing symptom burden.

Our study increases understanding of the role of symptom burden in asthma. Haselkorn and colleagues and Meltzer and colleagues both examined severe asthma populations to evaluate the prognostic value of symptom monitoring.^50^, ^51^ In Haselkorn and colleagues’ study, very poorly controlled asthma (defined by at least one uncontrolled domain across the mini-Asthma Quality-of-Life Questionnaire, reliever use, FEV_1_, and the Asthma Therapy Assessment Questionnaire) was associated with a six-times higher risk of one or more asthma attacks.^50^ In the study by Meltzer and colleagues, post-hoc analysis of an RCT population with severe asthma showed a 50% increased risk of one or more asthma attacks for each 1-point increase in ACQ-7 score, which incorporates reliever use and FEV_1_ in addition to the items examined by ACQ-5. Conversely, Schatz and colleagues studied a population spanning the full spectrum of asthma severity and reported more modest prognostic effects of symptom burden.^52^ Our ORACLE2 analysis of RCT control groups, adjusted for relevant clinical and biological factors, yielded a magnitude of risk similar to that found by Schatz and colleagues.^52^ None of these previous studies quantified the incremental value of symptoms in multivariable modelling or ascertained its use in identifying treatment-responsive patients—gaps the present study fills.

Symptom burden might have greater relevance when interpreted in context rather than in isolation. Importantly, because severe attacks were defined by oral corticosteroids prescription, and oral corticosteroid use is often prompted by symptoms without additional assessment, the prognostic value of baseline ACQ-5 scores is probably biased upwards. Even with that bias, ACQ-5 scores added little incremental explanatory power in multivariable modelling. This finding could be explained by the dissociation between patient-reported symptoms and underlying disease activity we observed, similar to what was previously reported in a landmark cluster analysis of primary care and severe asthma populations.^53^ The approximate 9% increment in asthma attack risk per 0·5-point increase in ACQ-5 indicates that symptom changes at the minimal clinically important difference translate into small changes in future risk; however, a 1·1-point increment was estimated to increase risk by a more clinically meaningful 20%. Crucially, the absence of theragnostic value of ACQ-5 score in predicting treatment-related reductions in asthma attacks is a novel observation, compared with the well established combined prognostic and theragnostic performance of type 2 biomarkers.^28^, ^29^, ^30^, ^31^ In our study, both relative and absolute treatment effects showed strong, graded associations with blood eosinophil counts and FeNO. Conversely, patients with low measurements of both biomarkers had little or no treatment benefit, despite many of the parent studies enriching for type 2 high populations.

These findings have practical implications. Although multimodal assessment is recommended by GINA,^1^ escalation based only on symptoms remains common. Given the toxicity of corticosteroids and the long-term costs of biologics, escalation decisions could benefit from assessment beyond symptoms alone. For example, type 2 biomarkers reflect targetable inflammatory pathways, whereas symptoms can reflect perception, airway mechanics, or comorbidities. Potentially, an increase in symptoms could be most useful to prompt objective assessment before escalation, analogous to what is done in other chronic diseases.^8^ When symptoms are associated with low type 2 biomarker measurements, emphasising multimodal approaches that address non-inflammatory drivers, such as smoking, obesity, anxiety, and breathing pattern disorder, could be important.

Strengths of our study include the multiplicity of study designs and clinical contexts analysed, for which a large individual patient data meta-analysis (ORACLE2^28^, ^34^) was leveraged. The breadth of analyses, contexts, and interventions enabled a detailed investigation of the value of symptom burden in asthma management. Our study of patients spanning the spectrum of asthma severities suggests that observations made on the prognostic value of ACQ-5 in patients severe asthma^50^ might be overestimated compared with its value in patients with milder forms. The analyses in studies recruiting people with asthma regardless of symptom burden (the ORACLE2 any-ACQ dataset,^35^, ^38^, ^39^, ^40^, ^41^ DREAM,^35^ and SA-OT^26^) are noteworthy and provide an additional layer of results to ascertain the value of symptoms.

Our results have several nuances. First, our estimation of the theragnostic value of ACQ-5 to predict reductions in asthma attacks was based on escalation to an anti-IL-5 biologic, ignoring the potential effect of anti-inflammatory reliever strategies. Both these interventions—a biologic and anti-inflammatory reliever—are widely efficacious for reducing risk and improving symptoms regardless of type 2 status.^39^, ^54^, ^55^ Second, we did not examine outcomes such as changes in ACQ-5 score or FEV_1_, endpoints known to be dissociated from attack risk reduction. For example, long-acting β-agonist monotherapy improves airflow and symptoms without reducing the risk of severe asthma attacks, and potentially increasing this risk.^56^, ^57^ Finally, asthma attacks in the PRISMA^33^ and BOOST^32^ studies were identified by acutely worsening symptoms, underscoring the importance of symptom monitoring in identifying opportunities for further evaluation.^51^

In addition to the nuances, our analysis has limitations. First, we did not consider symptom measures other than the ACQ-5; however, this score is widely adopted, notably in the inclusion criteria of most RCTs in asthma.^10^, ^11^, ^12^, ^13^, ^14^, ^15^, ^16^, ^17^, ^18^, ^19^, ^20^, ^21^, ^22^, ^23^ Second, the asthma attacks we analysed were classified as severe on the basis of oral corticosteroid prescription, yet we had no information on the degree of acute airflow limitation or the overall clinical severity of the episode. Similarly, our study populations were largely symptomatic by design, although we did analyse studies that had a large proportion of patients with symptom-controlled asthma (the ORACLE2 any-ACQ dataset^35^, ^38^, ^39^, ^40^, ^41^ and DREAM^35^) to support our observations. Third, only baseline ACQ-5 score and type 2 biomarkers were analysed, whereas fluctuations in these parameters can carry important information. Fourth, although both PRISMA and BOOST FEV_1_ predicted values were calculated with race-neutral equations,^58^ RCTs that were part of ORACLE2 probably applied variable prediction rules. Furthermore, analyses stratified by race were not done. Fifth, our conclusions are largely based on selected and highly adherent RCT populations; although we included observational cohorts, these analyses are associative and do not establish causality. Finally, this work represents retrospective analyses of existing trials and cohorts selected on the basis of permission to analyse the data, which might introduce selection bias. A prospective prespecified analysis of symptom-only versus multimodal therapeutic escalation in the context of a RCT would be required to definitively establish clinical utility.

In conclusion, across a large individual patient-level meta-analysis of 22 trials and complementary chronic and acute asthma cohorts, we observed that symptom burden showed modest incremental prognostic value for asthma attack risk and little alignment with inflammatory biology. By contrast, type 2 biomarkers more reliably identified patients at higher risk and those most likely to respond to treatment. Although symptoms are indispensable for recognising clinical deterioration, they might be insufficient on their own to guide anti-inflammatory escalation. Our findings support more individualised care, in which symptoms, attacks, and objective measures of airway pathology are interpreted together rather than in isolation, an approach that warrants prospective evaluation.

### Contributors

### Data sharing

Data underlying each trial are available according to the terms in the original trial publications. The aggregate dataset generated by this study can be shared if the proposed use of the data has been approved by all original data providers. Requests should be made to the corresponding authors via email. The analytical programming code is available on GitHub.

## Declaration of interests

SC has received non-restricted research grants from the National Institute for Health and Care Research Oxford Biomedical Research Centre, the Quebec Respiratory Health Research Network, the Association Pulmonaire du Québec, the Academy of Medical Sciences, AstraZeneca, bioMérieux, Circassia Niox Group, and Sanofi-Genyme-Regeneron; is the holder of the Association Pulmonaire du Québec's Research Chair in Respiratory Medicine and is a clinical research scholar of the Fonds de recherche du Québec; has received speaker honoraria from AstraZeneca, GlaxoSmithKline, Sanofi-Regeneron, Circassia Niox Group, and Valeo Pharma; has received consultancy fees from FirstThought, Apogee Therapeutics, Connectio biopharma, Upstream Bio, AstraZeneca, GlaxoSmithKline, Sanofi-Regeneron, Access Biotechnology and Access Industries; has received sponsorship to attend or speak at international scientific meetings by or for AstraZeneca and Sanofi-Regeneron; is an advisory board member and holds stock options for Biometry (a company developing a fractional exhaled nitric oxide device called myBiometry); is co-inventor for the patent filed as Method for alleviating dyspnoea with neuromodulation; advised the Institut national d’excellence en santé et services sociaux for an update of the asthma general practice information booklet for general practitioners as well as therapeutic indications for Enerzair; and is a member of the asthma steering committee of the Canadian Thoracic Society. CC-P received an education scholarship from the Université de Sherbrooke within the submitted work, and outside the submitted work, received speaker honoraria from AstraZeneca, GlaxoSmithKline, and Sanofi-Regeneron, and consultancy fees from AstraZeneca, GlaxoSmithKline, and Sanofi-Regeneron. PL reports an investigator-initiated unrestricted research grant from Sanofi-Regeneron for the submitted work; outside the submitted work, he reports consulting fees from AstraZeneca, GlaxoSmithKline, and Sanofi-Regeneron, and speaker honoraria from AstraZeneca, GlaxoSmithKline, Sanofi-Regeneron, Boehringer Ingelheim, and Novartis. LGH has received grant funding, participated in advisory boards, and given lectures at meetings supported by Amgen, AstraZeneca, Boehringer Ingelheim, Circassia, Hoffmann la Roche, GlaxoSmithKline, Novartis, Theravance, Evelo Biosciences, Sanofi, and Teva; has received grants from MedImmune, Novartis UK, Roche Genentech, Glaxo Smith Kline, Amgen, Astra Zeneca, MedImmune, Glaxo Smith Kline, Aerocrine, and Vitalograph; has received sponsorship for attending international scientific meetings from AstraZeneca, Boehringer Ingelheim, Chiesi, GSK, and Napp Pharmaceuticals; has taken part in asthma clinical trials sponsored by AstraZeneca, Boehringer Ingelheim, Hoffmann la Roche, and GlaxoSmithKline for which his institution received remuneration; and is the Academic Lead for the Medical Research Council Stratified Medicine UK Consortium in Severe Asthma, which involves industrial partnerships with a number of pharmaceutical companies including Amgen, AstraZeneca, Boehringer Ingelheim, GlaxoSmithKline, Hoffmann la Roche, and Janssen. PB has received research funding from Genentech via the University Hospitals of Leicester NHS Trust and DEShawResearch via the University of Leicester, consultancies for Genentech and DEShawResearch via the University of Leicester, one honorarium from AstraZeneca, and support to attend scientific meetings from GSK and Sanofi Genzyme. SR reports grants from the Charlies Research Foundation, the National Health and Medical Research Council, the Medical Research Future Fund, and the Raine Medical Research Foundation paid to his institution; consulting fees from Connect BioPharma; speaker honoraria from AstraZeneca, GlaxoSmithKline, Chiesi, Boehringer Ingelheim, and Sanofi; travel support from AstraZeneca, Boehringer Ingelheim, and Sanofi; and advisory board participation for GlaxoSmithKline, Sanofi-Regeneron, and AstraZeneca, outside the submitted work. MEW has received consulting, advisory, or speaking honoraria from Abbvie, Allakos, Amgen, Apogee, Areteia Therapeutics, Arrowhead Pharmaceutical, AstraZeneca, Avalo Therapeutics, Belenos Bio, Celldex, Connect Biopharma, Cytoreason, Eli Lilly, Enveda, Equillium, General Mediciens, Gilead, GlaxoSmithKline, Jasper, Kinaset, Kymera, Merck, MyBiometry, Pharming, Phylaxis, Pfizer, Pulmatrix, Rapt Therapeutics, recludix Pharma, Regeneron, Roche Genentech, Sanofi Genzyme, Sentien, Sound Biologics, SpiroMedical, Tetherex Pharmaceuticals, Uniquity Bio, Upstream Bio, Verona Pharma, and Zurabio. He is on the data safety and monitoring board for Sentien. He holds stock options for Upstream Bio and Cellergy. GB has received speaker honoraria from AstraZeneca, Boehringer Ingelheim, Chiesi, GSK, Merck Sharp & Dohme, Novartis, and Sanofi-Regeneron, and is past president of the Belgian Respiratory Society. SED received consultancy fees from AstraZeneca. CEB has received grants and consultancy fees from 4D Pharma, Areteia, AstraZeneca, Chiesi, Genentech, GSK, Mologic, Novartis, Regeneron Pharmaceuticals, Roche, and Sanofi. MC has received grants or contracts from American Lung Association, Apogee, AstraZeneca, Celldex, Connect BioPharma, Enveda, Gala Therapeutics, Genentech, GSK, Kymera, National Institutes of Health, Nocion, Novartis, PCORI, Pfizer, Pulmatrix, Sanofi-Aventis, Shionogi, Teva, and Theravance Biopharma. He has also received consulting fees from Allakos, Amgen, Apogee, Apreo Health, Arrowhead Pharmaceuticals, Blueprint Medicines, Connect BioPharma, Evommune, Genentech, Generate Biomedicines, GSK, Jasper, Johnson & Johnson, Kinaset, Merck, Novartis, OM Pharma, Pfizer, Pioneering Medicines, Sanofi-Aventis, Teva, Third Rock Ventures, Upstream Bio, Verona Pharmaceuticals, and VIDA; honoraria from Amgen, AstraZeneca, Med Learning Group, Regeneron Pharmaceuticals, and Sanofi; and stock options from Aer Therapeutics. NAH has received honoraria for serving as a consultant or advisor to GSK, AstraZeneca, Genentech, Sanofi, Regeneron, Verona, and Amgen and research grant support from GSK, AstraZeneca, Genentech, Regeneron, and Sanofi. DJJ has received advisory board and speakers’ fees from AstraZeneca, Boehringer Ingelheim, Novartis, Teva, GSK, Sanofi-Regeneron, and Chiesi. NM is an employee and shareholder of AstraZeneca. AM is an employee of GlaxoSmithKline. PH is an employee of GlaxoSmithKline and holds shares in GSK. MEH is an employee of Sanofi. RG is an employee of Regeneron and holds shares in the company. CTJH is a former employee of Genentech and no longer holds stock, and is a current employee of AbbVie and owns AbbVie stock. VD is an employee of Novartis. RWB has received institutional research funding from AstraZeneca, Teva, Health Research Council, Cure Kidz, and Perpetual Guardian; personal fees from AstraZeneca, Avillion, and Teva; and is chair of the Asthma Foundation of New Zealand adolescent and adult asthma guidelines, a reviewer for the Global Initiative for Asthma, and a former member of the Global Initiative for Chronic Obstructive Lung Disease board. JKS has received non-restricted research grants from AstraZeneca, European Respiratory Society Severe Heterogeneous Asthma Research collaboration—Patient Centred Clinical Research Collaboration, Register of Adult Patients with Severe Asthma for Optimal Disease Management Foundation, and ZonMw. EWS received consultancy fees from GSK and holds royalties from Springer for the book *Clinical Prediction Models*. IDP received speaker honoraria, advisory panel honoraria, educational event payments, and meeting sponsorship from AstraZeneca, Aretriea, Circassia, Amgen, Novartis, Chiesi, Sanofi-Regeneron, Menarini, GSK, Teva, Merck, and Genentech, and holds stock options for Upstream Bio and Mybiometry. All other authors declare no competing interests.
