# Supplementary material for: Symptom burden to characterise, predict, and prevent asthma attacks: a patient-level meta-analysis of randomised trials and translational prospective studies
Source: Lancet Respir Med. 2026 Oct;14(10):869–82. doi: 10.1016/S2213-2600(26)00150-5 (PMC13623818; doi:10.1016/S2213-2600(26)00150-5)
Supplement: Supplementary file 1 [file mmc1.pdf]

THE LANCET
Respiratory Medicine

Supplementary appendix

This appendix formed part of the original submission and has been peer reviewed. We post it as supplied by the authors.

Supplement to: Couillard S, Meulmeester FL, Charland P, et al. Symptom burden to characterise, predict, and prevent asthma attacks: a patient-level meta-analysis of randomised trials and translational prospective studies. *Lancet Respir Med* 2026; published online Aug 20. https://doi.org/10.1016/S2213-2600(26)00150-5.

EVALUATING SYMPTOM BURDEN TO CHARACTERISE, PREDICT, AND PREVENT ASTHMA ATTACKS: A PATIENT-LEVEL ANALYSIS OF RANDOMISED TRIALS AND TRANSLATIONAL PROSPECTIVE STUDIES

SUPPLEMENTARY APPENDIX

Table of Contents

Table of Contents 1

1. Supplementary Figures 2
   - FIGURE S1. Five item Asthma Control Questionnaire (ACQ) mean score and individual items prognostic value. 2
   - FIGURE S2. Five item Asthma Control Questionnaire (ACQ) mean score and individual items theragnostic value in DREAM. 3
   - FIGURE S3. Five item Asthma Control Questionnaire (ACQ) mean score and individual items theragnostic value in PRISMA. 4
2. Supplementary Tables 5
   - TABLE S1. Modified treatment step definitions for this study 5
   - TABLE S2. Study-level information for the trials that provided individual patient data 6
   - TABLE S3. Baseline characteristics of the ORACLE2 – Items subset 12
   - TABLE S4. Trial distribution of patients in the ORACLE2 study subsets 13
   - TABLE S5. Cytokine data distribution in SA-OT, PRISMA and BOOST studies 14
   - TABLE S6. Cross-sectional association between ACQ-5 and other asthma characteristics, per study: 16
   - TABLE S7. Cross-sectional association between ACQ-5 and other asthma characteristics, pooled analysis: 19
   - TABLE S8 ACQ-5 Association with Asthma Exacerbations 20
   - TABLE S9.ACQ-5 Increment Needed for Target Rate Ratios 21
   - TABLE S10. Adjusted rate ratios for ACQ-5 ordinal classification 22
   - TABLE S11 – Asthma attack prediction model performance 23
3. Supplementary References 24

1. Supplementary Figures

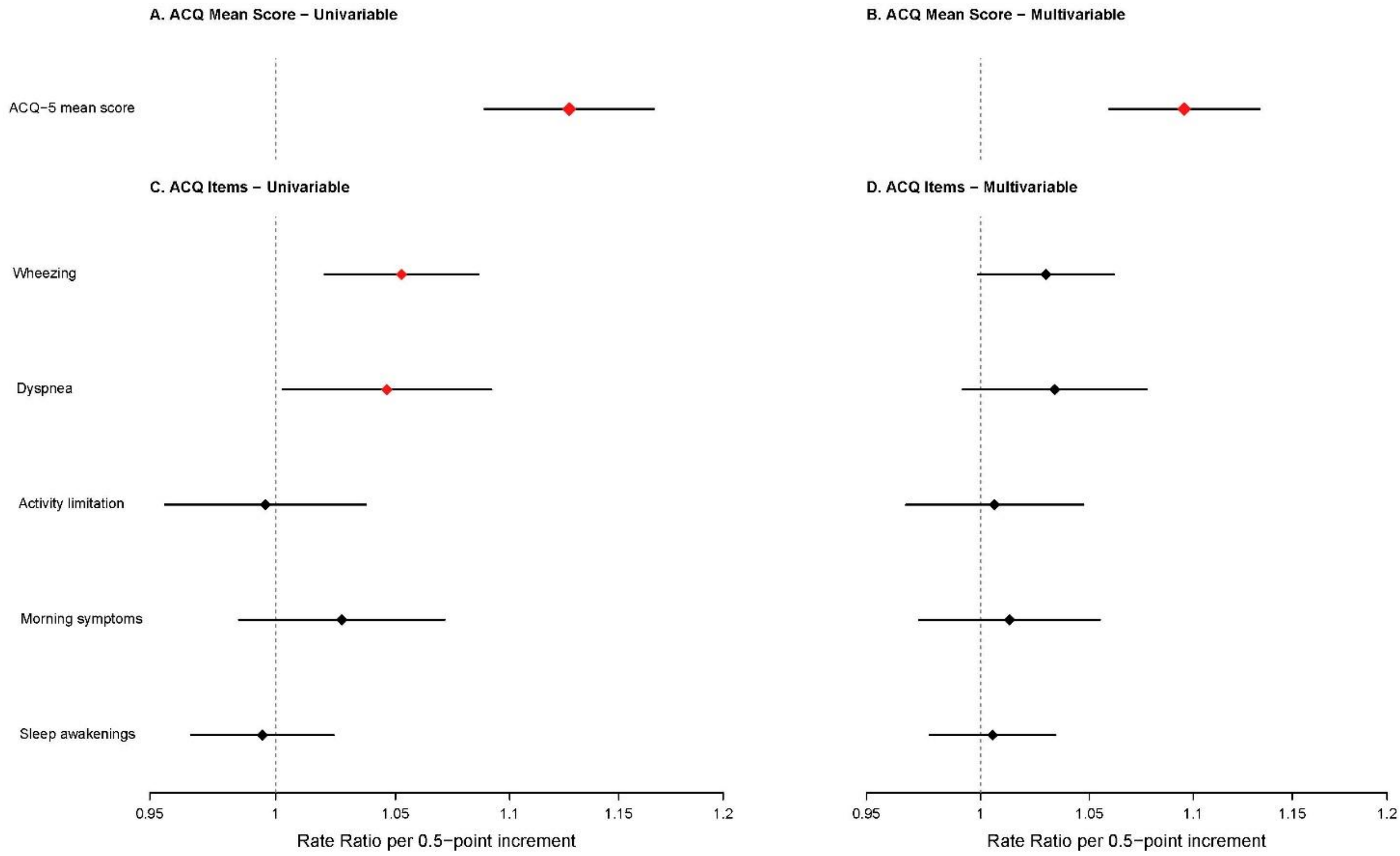

FIGURE S1. Five item Asthma Control Questionnaire (ACQ) mean score and individual items prognostic value.

Adjusted rate ratios per 0.5-point increment are shown in univariable (Panels A and C) and multivariable (Panels B and D) models. Analysis done in the ORACLE2-ACQ Items subset.

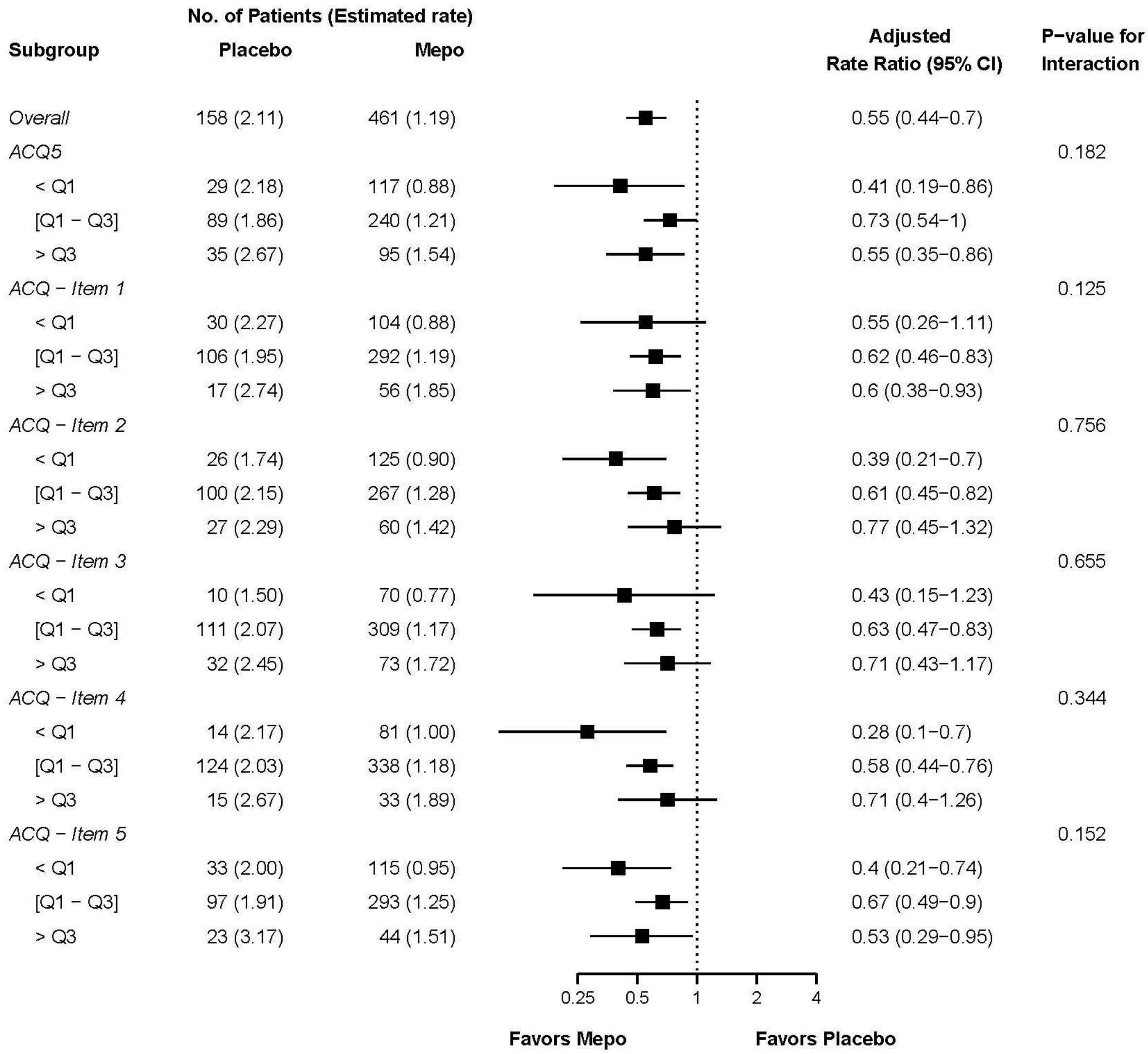

FIGURE S2. Five item Asthma Control Questionnaire (ACQ) mean score and individual items theragnostic value in DREAM.

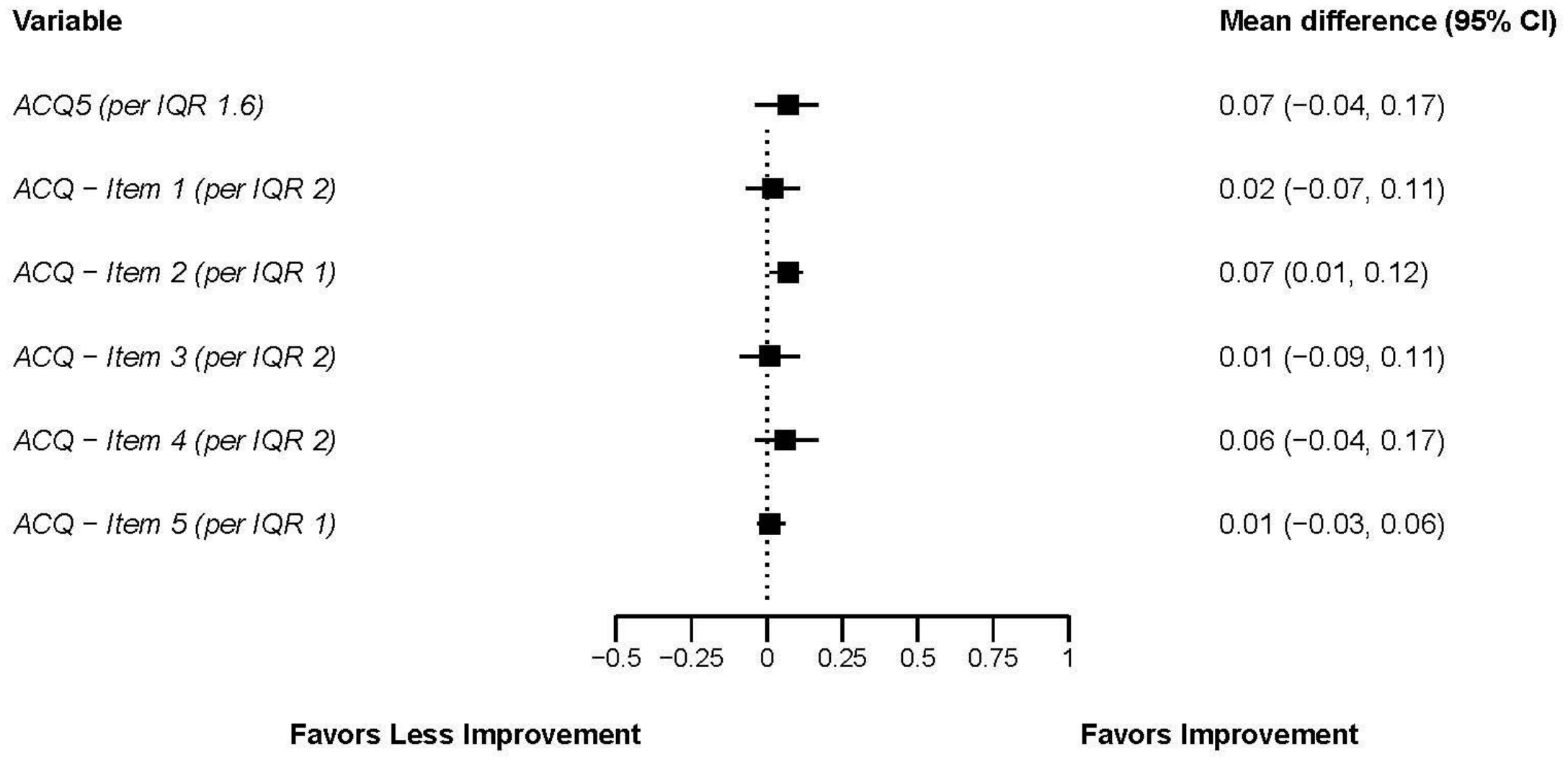

FIGURE S3. Five item Asthma Control Questionnaire (ACQ) mean score and individual items theragnostic value in PRISMA.

2. Supplementary Tables

TABLE S1. Modified treatment step definitions for this study

**Treatment step**	**Definition**
Step 1	As-needed short-acting beta2-agonist
Step 2	Daily low dose ICS or<br>As-needed low dose inhaled corticosteroid (ICS)-formoterol<br>Daily leukotriene receptor agonist
Step 3	Daily low dose ICS + an additional controller therapy
Step 4	Any medium dose ICS-containing regimen
Step 5	Any high dose ICS-containing regimen or<br>Any maintenance systemic corticosteroid use (defined as use of systemic corticosteroids for ≥50% of the previous year)

Modified from GINA 2017 and 2023 (Global Initiative for Asthma (GINA), 2023) guidelines.

TABLE S2. Study-level information for the trials that provided individual patient data

**Trial(s) reported** [registration no.]	**Study design**	**N trial randomised / n analysed**	**Key inclusion criteria**	**ACQ inclusion criteria**	**Recruitment biomarker targets**	**Endpoints**
**AZISAST (AZIthromycin in Severe ASThma)** [NCT00760838]	Randomised, placebo-controlled double-blind parallel-group	109 / 54	• Age 18–75<br>• GINA step 4 or 5<br>• High doses of ICS/LABA<br>• At least 2 severe asthma exacerbations or LRTI requiring antibiotics within the previous 12 months	-	All patients: FeNO level below the upper limit of normal according to gender, atopic status, and smoking history	Primary<br>• Severe asthma exacerbations and/or LRTI requiring antibiotics<br>Secondary<br>• FEV1, PEF, AQLQ and ACQ
**Benralizumab phase 2b** [NCT01238861]	Randomised, placebo-controlled, double-blind, parallel-group dose-ranging	609 / 211	• Aged 18–75<br>• Medium-dose high-dose ICS/LABA for at least 1 year<br>• History of two to six severe exacerbations in the past year<br>• FEV1 40%-90%, reversibility	• ACQ-6 score of 1.5 or higher	-	Primary<br>• Severe asthma annual exacerbation rate in eosinophilic individuals<br>Secondary<br>• Change from baseline in FEV1, ACQ-6, overall symptom score, and AQLQ
**BOOST (BreakthrOugh asthma attacks treated with Oral STeroids; substudy of the Oxford Airways Study)**	Single-centre, prospective cohort study (pilot)	60 / 60	• Aged ≥18 years<br>• Treated with an anti-IL5, anti-IL5Rα, or anti-IL4Rα monoclonal antibody for asthma, and on stable treatment for ≥2 months<br>• History of ≥1 severe exacerbations since starting mAb treatment	-	-	Primary<br>• Proportion of treatment failure at day 28 between FeNO-high (≥25 ppb) and FeNO-low (<25 ppb) groups.<br>Secondary<br>• Change from baseline in FEV1, ACQ-5, and total VAS symptoms across visits between FeNO-high and FeNO-low groups
**CAPTAIN (Clinical Study in Asthma Patients Receiving Triple Therapy in a Single Inhaler)** [NCT02924688]	Randomised, double-blind, parallel-group, phase 3A	2439 / 1218	• Aged ≥18 years<br>• ICS/LABA (>250 µg per day fluticasone or equivalent) for at least 12 consecutive weeks<br>• Documented health-care contact or temporary change in asthma therapy for acute asthma within 1 year<br>• FEV1 30%-85%, reversibility	• ACQ-6 ≥1.5	-	Primary<br>• Change from baseline in FEV1<br>Secondary<br>• Annualised rate of moderate and/or severe asthma exacerbations
**COSTA** [NCT01582503]	Randomised, placebo-	578 / 145	• Aged 18–75 years<br>• FEV1 40–80 %, reversibility	• ACQ-5 ≥1.5	All patients: At least one positive aero-	Primary outcome<br>• Annualised rate of severe asthma

	controlled double-blind, parallel-group, phase 2		• Daily use of ICS (≥400 µg/day total daily dose of FP) and second controller for 3 months<br>• History of at least one severe asthma exacerbation in the 18 months prior		allergen or a total serum IgE ≥75 IU/mL	exacerbations<br>Secondary outcomes<br>• FEV1 and the change in asthma symptoms
**Trial(s) reported** *continued*	**Study design**	**N trial randomised / n analysed**	**Key inclusion criteria**	**ACQ inclusion criteria**	**Recruitment biomarker targets**	**Endpoints**
**DREAM (Dose Ranging Efficacy And safety with Mepolizumab)** [NCT01000506]	Randomised, placebo-controlled, double-blind, parallel-group, dose-ranging	621 / 155	• Age 12–74 years<br>• History of two or more severe exacerbations<br>• FEV1 <80%<br>• At least 880 µg fluticasone propionate/day, with or without OCS.	-	All patients, past 12 months, 1 of:<br>i) sputum eosinophil count ≥3%,<br>ii) FENO ≥50 ppb,<br>iii) blood eosinophil ≥0·3×10⁹/L.	Primary outcome<br>• Rate of severe clinically significant asthma exacerbations<br>Secondary outcomes<br>• Rate of exacerbations, blood and sputum eosinophil counts, FEV1, AQLQ and ACQ
**Dupilumab phase 2b** [NCT01854047]	Randomised, placebo-controlled, double-blind, parallel-group, dose-ranging	769 / 155	• Adults (aged ≥18 years)<br>• Treatment with medium-to-high-dose ICS/LABA at least 1 month<br>• FEV1 of 40–80%<br>• At least one severe asthma exacerbation	• ACQ-5 ≥1.5	-	Primary endpoint<br>• Change in FEV1 in patients with baseline blood eosinophil ≥300<br>Secondary endpoints<br>• Change from baseline in FEV1; annualised severe asthma exacerbation rate; ACQ-5, AQLQ, FeNO
**EXTRA** [NCT00314574]	Randomised, placebo-controlled double-blind, parallel-group,	850 / 420	• Aged 12 to 75 years<br>• History of severe allergic asthma for at least 1 year<br>• FEV1 of 40-80%<br>•	• Asthma was not well-controlled despite treatment with high-dose ICS/LABA with or without other controllers (including OCS), based on NAEPP guidelines. Asthma was considered not well-controlled if patients had persistent asthma	All patients, past 12 months:<br>At least one positive aero-allergen or a total serum IgE 30-<700 IU/mL	Primary end point<br>• Rate of severe asthma exacerbations<br>Secondary end points<br>• Change in total asthma symptom severity score (TASS), change in mean puffs per day of albuterol; and change AQLQ

Trial(s) reported *continued*	Study design	N trial randomised / n analysed	Key inclusion criteria	ACQ inclusion criteria	Recruitment biomarker targets	Endpoints
				symptoms with current therapy.		
**LAVOLTA I, II** [NCT01867125, NCT01868061]	Randomised, placebo-controlled, double-blind, parallel-group, replicate, phase 3,	I: 1081 / 362<br>II: 1067 / 354	• Aged 18–75 years<br>• FEV1 40–80%<br>• ICS (500–2000 µg per day fluticasone propionate or equivalent) for at least 6 months and at least one additional controller medication	• ACQ-5 ≥1.5	-	Primary endpoint<br>• Rate of severe asthma exacerbations in biomarker-high<br>Secondary endpoints<br>• Absolute changeFEV1; time to first asthma exacerbation; AQLQ, ACQ-5
**LUSTER I, II** [NCT02555683, NCT02563067]	Randomised, placebo-controlled, double-blind, parallel-group, replicate, phase 3	I: 894 / 298<br>II: 877 / 287	• Aged 12 years or older<br>• Uncontrolled asthma on dual/triple asthma therapy with medium/high dose ICS with up to two controllers with or without OCS<br>• History of two or more asthma exacerbations within the previous 12 months.<br>• FEV1 ≤80%	• ACQ-5 ≥1.5	• 2/3 : blood eosinophil count $\geq 0{\cdot}25\times10^9$/L<br>• 1/3 : blood eosinophil count $<0{\cdot}25\times10^9$/L.	Primary endpoint<br>• Number of moderate to severe asthma exacerbations per patient year both in patients with high blood eosinophil counts (≥250 cells/µL) and in all patients<br>Secondary endpoints<br>• Change from baseline in FEV1, ACQ-5, AQLQ
**LUTE, VERSE** [NCT01545440, NCT01545453]	Randomised, placebo-controlled double-blind, replicate studies, phase IIb. Amended protocol and early termination	L: 258 / 66<br>V: 205 / 50	• Patients aged 18–75 years<br>• Daily use of 500–2000 µg/day of fluticasone and a second asthma controller medication ≥12 months<br>• FEV1 40–80%	• ACQ-5 ≥1.5	-	Primary endpoint<br>• Rate of severe asthma exacerbations<br>Secondary endpoints<br>• Change in FEV1 from baseline, time to first exacerbation, AQLQ
**MILLY** [NCT00930163]	Randomised, placebo-controlled double-blind, parallel-group	219 / 112	• Age 18-65 years<br>• For at least 6 months of ICS (≥200 and ≤1000 µg of inhaled fluticasone propionate daily)<br>• FEV1 40%-80%,	• ACQ-5 ≥1.5	-	Primary outcome<br>• Relative change in FEV1<br>Secondary outcomes<br>• Rates of moderate and severe exacerbations, PEF, ACQ5, ACDD, and use of rescue medication
**NAVIGATOR** [NCT03347279]	Randomised, placebo-controlled	1061 / 517	• Age 12-80 years<br>• Medium/high-dose ICS for at least 12 months and at least one additional controller, with or without oral	• ACQ-6 ≥1.5	• 50% : blood eosinophil count $\geq 0{\cdot}3\times10^9$/L	Primary endpoint<br>• Rate of severe asthma exacerbations<br>Secondary endpoints

	double-blind parallel-group, phase 3		glucocorticoids, for at least 3 months before<br>• FEV1 <80%<br>• At least two exacerbations in the 12 months before		• 50% : blood eosinophil count $<0{\cdot}3\times10^9$/L.	• Change in FEV1 from baseline, time to first exacerbation, AQLQ
**Trial(s) reported** *continued*	**Study design**	**N trial randomised / n analysed**	**Key inclusion criteria**	**ACQ inclusion criteria**	**Recruitment biomarker targets**	**Endpoints**
**NOVEL START** [ACTRN12615 000999538]	Randomised, open-label, controlled parallel group trial	675 / 218	• Age 18 to 75 years<br>• SABA as the sole asthma therapy in the previous 3 months on at least two occasions, but on an average of two or fewer occasions per day in the previous 4 weeks	-	-	Primary outcome<br>• Annualised rate of asthma severe/moderate exacerbations<br>Secondary outcomes<br>• Number of exacerbations, number of severe exacerbations, ACQ-5, FeNO, oral prednisone use
**PACT** [NCT00272506]	Randomised, placebo-controlled, double-blind, parallel group	285 / 16	• Age 6-14 years<br>• FEV1 ≥70% predicted<br>• Mild-moderate persistent asthma	-	-	Primary outcome<br>• Percent of asthma control days<br>Secondary analyses<br>• Percent of episode-free days, number of severe exacerbations and time to the first exacerbation, ACQ
**PATHWAY** [NCT02054130]	Randomised, placebo-controlled double-blind, parallel group	550 / 133	• Age 18 to 75<br>• Asthma not well controlled despite treatment with medium/high dose LABA/ICS at least 6 months<br>• At least two severe asthma exacerbations<br>• FEV1 40%-80%	• ACQ-6 ≥1.5	• ≥50% : blood eosinophil count	Primary endpoint<br>• Annualised rate of severe asthma exacerbations Secondary endpoints<br>• Changes FEV1, ACQ-6, AQLQ, asthma symptom score, and FVC, annualized rate of exacerbations, time to the first exacerbation
**PRACTICAL** [ACTRN12616 000377437]	Randomised, open-label, controlled parallel-group, pragmatic trial	890 / 307	• Aged 18 to 75<br>• SABA reliever alone in the past 12 weeks, symptoms or need for SABA on ≥2 occasions in the past 4 weeks, or a history of a severe asthma exacerbation in the past 52 weeks<br>• SABA reliever with low/moderate doses of ICS in the past 12 weeks, partly or well controlled or uncontrolled asthma with poor adherence	-	-	Primary outcome<br>• Number of severe asthma exacerbations per patient per year<br>Secondary outcomes<br>• Proportion of patients with a least one severe exacerbation; time to first exacerbation; ACQ-5, FEV1, FeNO

Trial(s) reported *continued*	Study design	N trial randomised / n analysed	Key inclusion criteria	ACQ inclusion criteria	Recruitment biomarker targets	Endpoints
**PRISMA** [NCT05870215]	Single-centre, prospective cohort study (pilot)	**53 /53**	• Age 12 years or older<br>• Physician-diagnosed asthma >6 months with objective confirmation (GINA criteria)<br>• Experiencing a severe asthma attack	**-**	-	Primary outcome<br>• Change in post-BD FEV1 after 7 days of oral corticosteroids; patients stratified T2-Low/Low, BEC <0.15×10^9 cells/L and FeNO <25 ppb; T2-High/High, blood eosinophils ≥0.30×10^9 cells/L and FeNO ≥35 ppb and T2-Mid, not meeting Low/Low-High/High criteria<br>Secondary outcomes<br>• Change in ACQ-5, and the proportion of patients achieving a MCID ≥0.1L for post-BD FEV1 or ≥0.5 points for ACQ-5
**QUEST** [NCT02414854]	Randomised, placebo-controlled, double-blind, parallel-group, phase 3	1902 / 634	• Age 12 years or older<br>• Medium/high-dose ICS/LABA plus up to 2 controllers<br>• FEV1 80% or less, reversibility<br>• Severe asthma exacerbation in the previous year	• ACQ-5 ≥1.5	**-**	Primary efficacy end points<br>• Annualised rate of severe exacerbation events and the absolute change from baseline in the FEV1<br>Secondary end points<br>• Percentage change from baseline in the FEV1
**SA-OT (Severe asthma on optimal therapy)**	Pooled cross-sectional of two cohorts (Oxford FeNO suppression cohort and RASP-UK bronchoscopy cohort)	74 /74	• Aged ≥18 years<br>• Severe asthma on high-dose ICS and/or maintenance OCS<br>• Confirmed treatment adherence through: 1) FeNO suppression testing or 2) participation in biomarker-guided management trial (RASP-UK) with objective adherence measures<br>• Availability of induced sputum samples for analysis	**-**	-	Primary outcome<br>• Association between FeNO and blood eosinophils with airway (sputum) and systemic (serum) inflammatory mediators (type-2 cytokines, chemokines, alarmins)<br>Secondary outcomes<br>• Trends in sputum and serum analytes across FeNO and blood eosinophil strata<br>• Correlations between FeNO, blood eosinophils, and inflammatory mediators<br>• FEV1, FEV1/FVC, ACQ-5, exacerbation frequency

**STRATOS I, II** [NCT02161757, NCT02194699]	Randomised, placebo-controlled, double-blind, parallel-group, replicate trials, phase 3	I: 1207 / 388<br>II: 856 / 413	• Aged 12–75 years<br>• Medium/high dose ICS/LABA for at least 3 months<br>• Additional controller medications if required<br>• ≥2 severe asthma exacerbation in the previous year	• ACQ-6 ≥1.5	**-**	Primary endpoint<br>• Annualised severe asthma exacerbation rate<br>Secondary endpoints<br>• FEV1, ACQ-6, AQLQ, time to first exacerbation; proportion of participants with one or more exacerbations

BD, bronchodilator; BPT, bronchial provocation test; ACQ, asthma control questionnaire; AQLQ, asthma quality of life questionnaire; FEV_1, forced expiratory volume in 1st second; FVC, forced vital capacity; ICS, inhaled corticosteroid; LABA, long-acting beta-agonist; LAMA, long-acting muscarinic antagonist; MCID, minimal clinically important difference; OCS, oral corticosteroid; PEF, peak expiratory flow.

TABLE S3. Baseline characteristics of the ORACLE2 – Items subset

Variable	Level	Value
n		3858
Age		48.96 (14.45)
Sex	Female	2442 (63.3)
	Male	1416 (36.7)
BMI (kg/m2)		28.58 (5.79)
Smoking	Never	3083 (79.9)
	Yes (current or ex)	775 (20.1)
Treatment Step	Step 3	843 (21.9)
	Step 1	226 (5.9)
	Step 2	315 (8.2)
	Step 4	1207 (31.3)
	Step 5	1267 (32.8)
Severe attacks in previous 12 months		1.62 (1.63)
FEV1 preBD % predicted		67.08 (16.18)
FEV1 reversibility postBD %		20.18 (19.79)
FEV1/FVC ratio		64.10 (12.17)
ACQ-5 baseline mean score		2.28 (1.05)
Blood eosinophil count, $x10^9/L$		0.24 [0.14, 0.40]
FeNO, ppb		22.33 [13.80, 40.00]
CRSsNP, yes/no	No	3344 (86.7)
	Yes	514 (13.3)
CRSwNP, yes/no	No	2562 (66.4)
	Yes	1296 (33.6)
Allergic rhinits	No	2484 (64.4)
	Yes	1374 (35.6)
Total severe asthma attacks during follow-up		2452.00 (0.00)
Follow-up, person*years		3141.84 (0.00)
Unadjusted annual rate		0.78 (0.00)

Numbers are mean (SD, n (%), or median [interquartile range]. BMI, body mass index; FEV1, forced expiratory volume in 1st second; FVC, forced vital capacity; FeNO, fractional exhaled nitric oxide; CRSsNP, chronic rhinosinusitis without nasal polyposis; CRSwNP, CRS with NP.

TABLE S4. Trial distribution of patients in the ORACLE2 study subsets

Enrolled Trial name	ORACLE2 – Full (N=6513)	ORACLE2 – Any ACQ (n=750)	ORACLE2 – Items (N=3858)
AZISAST[1]	54 (0.8%)	54 (7.2%)	54 (1.4%)
BENRAP2B[2]	211 (3.2%)	-	211 (5.5%)
CAPTAIN[3]	1218 (18.7%)	-	1218 (31.6%)
COSTA[4]	145 (2.2%)	-	-
DREAM[5]	155 (2.4%)	155 (20.7%)	155 (4.0%)
Dupilumab phase 2b[6]	155 (2.4%)	-	-
EXTRA[7]	420 (6.4%)	-	-
LAVOLTA 1[8]	362 (5.6%)	-	-
LAVOLTA 2[8]	354 (5.4%)	-	-
LUSTER 1[9]	298 (4.6%)	-	-
LUSTER 2[9]	287 (4.4%)	-	-
LUTE[10]	66 (1.0%)	-	66 (1.7%)
MILLY[11]	112 (1.7%)	-	112 (2.9%)
NAVIGATOR[12]	517 (7.9%)	-	517 (13.4%)
Novel START[13]	218 (3.3%)	218 (29.1%)	218 (5.7%)
PACT[14]	16 (0.2%)	16 (2.1%)	16 (0.4%)
PATHWAY[15]	133 (2.0%)	-	133 (3.4%)
PRACTICAL[16]	307 (4.7%)	307 (40.9%)	307 (8.0%)
QUEST[17]	634 (9.7%)	-	-
STRATOS 1[18]	388 (6.0%)	-	388 (10.1%)
STRATOS 2[18]	413 (6.3%)	-	413 (10.7%)
VERSE[10]	50 (0.8%)	-	50 (1.3%)

Detailed study characteristics are available in the quoted references, and an ORACLE2-specific per-trial participant data description is available in Meulmeester *et al.* (2025).[19]

TABLE S5. Cytokine data distribution in SA-OT, PRISMA and BOOST studies

Variable	BOOST (N=60)	FeNO+RASP-UK (N=74)	Prisma (N=53)	P-value
Sputum eosinophils (%)				
Median [Q1-Q3]	0 [0-2.13]	3.25 [0.750-19.1]	1.60 [0.275-11.3]	<0.001
Missing	8 (13.3%)	25 (33.8%)	5 (9.4%)	
Sputum eotaxin-3 (pg/mL)				
Median [Q1-Q3]	NA [NA-NA]	133 [23.9-366]	26.7 [4.67-104]	0.00398
Missing	60 (100%)	18 (24.3%)	3 (5.7%)	
Sputum IFN-gamma (pg/mL)				
Median [Q1-Q3]	NA [NA-NA]	0.395 [0.171-1.47]	4.70 [1.69-58.0]	<0.001
Missing	60 (100%)	18 (24.3%)	3 (5.7%)	
Sputum IL-13 (pg/mL)				
Median [Q1-Q3]	NA [NA-NA]	7.29 [6.03-11.6]	0.697 [0.655-1.34]	<0.001
Missing	60 (100%)	14 (18.9%)	4 (7.5%)	
Sputum IL-4 (pg/mL)				
Median [Q1-Q3]	NA [NA-NA]	0.302 [0.104-0.960]	0.0629 [0.0524-0.216]	<0.001
Missing	60 (100%)	14 (18.9%)	4 (7.5%)	
Sputum IL-5 (pg/mL)				
Median [Q1-Q3]	NA [NA-NA]	4.67 [1.72-11.4]	2.92 [0.542-11.9]	0.27
Missing	60 (100%)	14 (18.9%)	4 (7.5%)	
Sputum IL-33 (pg/mL)				
Median [Q1-Q3]	NA [NA-NA]	1.09 [0.296-2.19]	1.52 [1.02-2.42]	0.0409
Missing	60 (100%)	14 (18.9%)	4 (7.5%)	
Sputum TARC				
Median [Q1-Q3]	NA [NA-NA]	36.1 [17.2-91.8]	27.6 [8.74-64.2]	0.119
Missing	60 (100%)	18 (24.3%)	4 (7.5%)	
Sputum TNF-alpha (pg/mL)				

Variable	BOOST (N=60)	FeNO+RASP-UK (N=74)	Prisma (N=53)	P-value
Median [Q1-Q3]	NA [NA-NA]	2.55 [0.797-7.29]	1.29 [0.373-2.31]	0.0114
Missing	60 (100%)	18 (24.3%)	5 (9.4%)	
Sputum TSLP (pg/mL)				
Median [Q1-Q3]	NA [NA-NA]	6.69 [2.40-13.0]	4.31 [1.32-7.59]	0.0733
Missing	60 (100%)	14 (18.9%)	4 (7.5%)	
Sputum PGD2 (pg/mL)				
Median [Q1-Q3]	NA [NA-NA]	222 [133-404]	143 [43.3-299]	0.015
Missing	60 (100%)	18 (24.3%)	8 (15.1%)	

IFN, interferon; IL, interleukin; TARC, thymuc activation regulatory cytokine; TNF, tumour necrosis factor; TSLP, thymic stromal lymphopoietin; PGD2, prostaglandin D2. Cytokines were measured in phosphate buffered saline (PBS)-eluted sputum supernatant using electrochemiluminescence (MesoScale Discovery, USA), except for PGD2 which was measured by ELISA (Cayman Chemical, USA), in methods which were previously reported.[20–22] P-values are from nonparametric Wilcoxon rank-sum (2 groups) or Kruskall-Wallis tests (3 groups).

TABLE S6. Cross-sectional association between ACQ-5 and other asthma characteristics, per study:

Variables	Model	Estimate	CI	P_value	N analysed	N missing
Age (per 10 years)	ORACLE2 - Any-ACQ	-0.13	-0.18 to -0.09	0.000	750	0
Sex - Female	ORACLE2 - Any-ACQ	0.15	0.02 to 0.27	0.022	750	0
BMI (per 5)	ORACLE2 - Any-ACQ	0.02	0.01 to 0.03	0.000	750	0
GINA step	ORACLE2 - Any-ACQ	-0.47	-1.3 to 0.36	0.266	750	0
Blood eos (per 10-fold)	ORACLE2 - Any-ACQ	0.03	-0.16 to 0.22	0.746	750	0
FeNO (per 10-fold)	ORACLE2 - Any-ACQ	0.11	-0.08 to 0.31	0.262	750	0
FEV1% preBD (per 10% decrease)	ORACLE2 - Any-ACQ	0.04	-0.01 to 0.09	0.136	750	0
FEV1/FVC preBD (per 10% decrease)	ORACLE2 - Any-ACQ	0.01	-0.07 to 0.09	0.765	750	0
Previous asthma attack	ORACLE2 - Any-ACQ	-0.03	-0.26 to 0.2	0.809	750	0
Smoking status	ORACLE2 - Any-ACQ	0.18	0.06 to 0.29	0.003	750	0
Allergic Rhinitis	ORACLE2 - Any-ACQ	-0.02	-0.22 to 0.19	0.869	750	0
CRSsNP	ORACLE2 - Any-ACQ	-0.06	-0.28 to 0.15	0.551	750	0
CRSwNP	ORACLE2 - Any-ACQ	-0.15	-0.34 to 0.04	0.118	750	0
Age (per 10 years)	Severe Asthma	-0.04	-0.27 to 0.19	0.708	74	0
Sex - Female	Severe Asthma	0.41	-0.17 to 0.98	0.164	74	0
BMI (per 5)	Severe Asthma	-0.02	-0.26 to 0.21	0.853	74	0
Blood eos (per 10-fold)	Severe Asthma	-0.50	-2.08 to 1.08	0.530	74	0
FeNO (per 10-fold)	Severe Asthma	0.88	0 to 1.76	0.051	74	0
FEV1% preBD (per 10% decrease)	Severe Asthma	0.02	-0.14 to 0.17	0.825	74	0
FEV1/FVC preBD (per 10% decrease)	Severe Asthma	-0.07	-0.42 to 0.28	0.692	74	0
Previous asthma attack	Severe Asthma	-0.24	-0.83 to 0.35	0.420	74	0
Smoking status	Severe Asthma	0.16	-0.5 to 0.81	0.639	74	0
Sputum eosinophils (%), per 10-fold	Severe Asthma	0.26	-0.3 to 0.82	0.359	49	25
Sputum eotaxin-3 (pg/mL), per 10-fold	Severe Asthma	-0.06	-0.47 to 0.36	0.786	56	18
Sputum IFN-gamma (pg/mL), per 10-fold	Severe Asthma	-0.03	-0.62 to 0.57	0.932	56	18
Sputum IL-13 (pg/mL), per 10-fold	Severe Asthma	0.16	-0.87 to 1.2	0.751	60	14
Sputum IL-4 (pg/mL), per 10-fold	Severe Asthma	-0.37	-1.13 to 0.38	0.322	60	14

Sputum IL-5 (pg/mL), per 10-fold	Severe Asthma	-0.14	-0.9 to 0.63	0.721	60	14
Sputum IL-33 (pg/mL), per 10-fold	Severe Asthma	-0.02	-0.49 to 0.46	0.940	60	14
Sputum TARC (pg/mL), per 10-fold	Severe Asthma	0.03	-0.47 to 0.54	0.897	56	18
Sputum TNF-alpha (pg/mL), per 10-fold	Severe Asthma	0.09	-0.35 to 0.53	0.686	56	18
Sputum TSLP (pg/mL), per 10-fold	Severe Asthma	-0.03	-0.64 to 0.58	0.927	60	14
Sputum PGD2 (pg/mL), per 10-fold	Severe Asthma	-0.14	-0.91 to 0.63	0.719	56	18
Age (per 10 years)	PRISMA	0.01	-0.18 to 0.21	0.884	53	0
Sex - Female	PRISMA	0.38	-0.29 to 1.04	0.260	53	0
BMI (per 5)	PRISMA	0.23	0.06 to 0.41	0.011	53	0
GINA step	PRISMA	-0.56	-2.98 to 1.87	0.645	53	0
Blood eos (per 10-fold)	PRISMA	0.05	-0.9 to 1.01	0.909	53	0
FeNO (per 10-fold)	PRISMA	-0.80	-1.75 to 0.15	0.099	53	0
FEV1% preBD (per 10% decrease)	PRISMA	0.14	-0.06 to 0.34	0.161	53	0
FEV1/FVC preBD (per 10% decrease)	PRISMA	-0.17	-0.62 to 0.27	0.437	53	0
Previous asthma attack	PRISMA	-0.08	-0.77 to 0.61	0.820	53	0
Smoking status	PRISMA	0.30	-0.44 to 1.03	0.418	53	0
Allergic Rhinitis	PRISMA	0.41	-0.21 to 1.03	0.188	53	0
CRSsNP	PRISMA	0.15	-0.53 to 0.83	0.664	53	0
CRSwNP	PRISMA	0.66	-0.08 to 1.4	0.080	53	0
Sputum eosinophils (%), per 10-fold	PRISMA	-0.09	-0.65 to 0.46	0.741	48	5
Sputum eotaxin-3 (pg/mL), per 10-fold	PRISMA	0.13	-0.36 to 0.61	0.602	50	3
Sputum IFN-gamma (pg/mL), per 10-fold	PRISMA	-0.03	-0.29 to 0.23	0.822	50	3
Sputum IL-13 (pg/mL), per 10-fold	PRISMA	0.62	-0.54 to 1.77	0.289	49	4
Sputum IL-4 (pg/mL), per 10-fold	PRISMA	0.30	-0.29 to 0.89	0.315	49	4
Sputum IL-5 (pg/mL), per 10-fold	PRISMA	0.01	-0.46 to 0.47	0.981	49	4
Sputum IL-33 (pg/mL), per 10-fold	PRISMA	0.05	-0.67 to 0.77	0.896	49	4
Sputum TARC (pg/mL), per 10-fold	PRISMA	-0.43	-0.96 to 0.09	0.102	49	4
Sputum TNF-alpha (pg/mL), per 10-fold	PRISMA	0.02	-0.5 to 0.54	0.928	48	5
Sputum TSLP (pg/mL), per 10-fold	PRISMA	0.06	-0.39 to 0.52	0.774	49	4

Sputum PGD2 (pg/mL), per 10-fold	PRISMA	-0.35	-0.72 to 0.02	0.064	45	8
Age (per 10 years)	BOOST	-0.22	-0.42 to -0.02	0.032	60	0
Sex - Female	BOOST	0.60	0.07 to 1.14	0.028	60	0
BMI (per 5)	BOOST	0.08	-0.08 to 0.23	0.317	60	0
Blood eos (per 10-fold)	BOOST	2.83	-0.49 to 6.16	0.094	60	0
FeNO (per 10-fold)	BOOST	-0.15	-0.75 to 0.46	0.625	60	0
FEV1% preBD (per 10% decrease)	BOOST	0.12	0 to 0.25	0.050	60	0
FEV1/FVC preBD (per 10% decrease)	BOOST	-0.26	-0.52 to 0	0.052	60	0
Previous asthma attack	BOOST	0.51	-0.04 to 1.06	0.067	60	0
Smoking status	BOOST	-0.09	-0.68 to 0.49	0.748	60	0
Allergic Rhinitis	BOOST	-0.17	-0.96 to 0.61	0.659	60	0
CRSsNP	BOOST	0.32	-1.75 to 2.39	0.760	60	0
CRSwNP	BOOST	-0.06	-0.72 to 0.6	0.848	60	0
Sputum eosinophils (%), per 10-fold	BOOST	-0.20	-0.66 to 0.26	0.383	52	8

TABLE S7. Cross-sectional association between ACQ-5 and other asthma characteristics, pooled analysis:

Variables	No. of studies (k)	No. of participants	Estimate	CI lower bound	CI upper bound	P_value	I^2 (%)	Tau^2
Age (per 10 years)	4	937	-0.12	-0.17	-0.08	**<0.001**	0	0.000
Sex - Female	4	937	0.28	0.04	0.53	**0.022**	31	0.022
BMI (per 5)	4	937	0.06	-0.03	0.16	0.170	51	0.004
GINA step	2	803	-0.07	-0.45	0.32	0.734	17	0.022
Blood eos (per 10-fold)	4	937	0.03	-0.15	0.22	0.732	0	0.000
FeNO (per 10-fold)	4	937	0.03	-0.45	0.52	0.894	63	0.144
FEV1% preBD (per 10% decrease)	4	937	0.05	0.01	0.10	0.016	0	0.000
FEV1/FVC preBD (per 10% decrease)	4	937	-0.08	-0.24	0.07	0.308	39	0.010
Previous asthma attack	4	937	0.02	-0.22	0.27	0.844	18	0.013
Smoking status	4	937	0.17	0.06	0.28	**0.002**	0	0.000
Allergic Rhinitis	3	863	0.01	-0.18	0.20	0.909	0	0.000
CRSsNP	3	863	-0.04	-0.24	0.17	0.718	0	0.000
CRSwNP	3	863	0.05	-0.38	0.48	0.833	54	0.081
Sputum eosinophils (%), per 10-fold	3	149	-0.04	-0.34	0.26	0.801	0	0.000
Sputum eotaxin-3 (pg/mL), per 10-fold	2	106	0.02	-0.29	0.33	0.898	0	0.000
Sputum IFN-gamma (pg/mL), per 10-fold	2	106	-0.03	-0.27	0.21	0.814	0	0.000
Sputum IL-13 (pg/mL), per 10-fold	2	109	0.36	-0.41	1.14	0.354	0	0.000
Sputum IL-4 (pg/mL), per 10-fold	2	109	0.14	-0.33	0.60	0.571	0	0.000
Sputum IL-5 (pg/mL), per 10-fold	2	109	-0.01	-0.34	0.32	0.973	0	0.000
Sputum IL-33 (pg/mL), per 10-fold	2	109	-0.16	-0.68	0.37	0.559	0	0.000
Sputum TARC (pg/mL), per 10-fold	2	105	-0.20	-0.65	0.26	0.402	37	0.040
Sputum TNF-alpha (pg/mL), per 10-fold	2	104	0.06	-0.27	0.40	0.719	0	0.000
Sputum TSLP (pg/mL), per 10-fold	2	109	0.03	-0.33	0.39	0.863	0	0.000
Sputum PGD2 (pg/mL), per 10-fold	2	101	-0.31	-0.65	0.02	0.070	0	0.000

TABLE S8 ACQ-5 Association with Asthma Exacerbations

Cohort	Model	aRR per 1 unit	aRR per 0.5 unit	aRR per IQR	P-value
Full ORACLE2	Univariable*	1.268 [1.203-1.336]	1.126 [1.097-1.156]	1.330 [1.249-1.416]	< .0001
Full ORACLE2	Multivariable**	1.189 [1.129-1.252]	1.090 [1.063-1.119]	1.231 [1.157-1.309]	<.0001
Any ACQ Subset	Univariable	1.251 [1.095-1.428]	1.118 [1.046-1.195]	1.368 [1.136-1.647]	0.001
Any ACQ Subset	Multivariable**	1.180 [1.035-1.345]	1.086 [1.017-1.160]	1.261 [1.049-1.515]	0.014

*Univariable covariates: ACQ + trial adjustment only. **Multivariable covariates: adjusted for ACQ-5, sex, BMI, severe attack history in preceding 12 months (yes/no), GINA treatment step, FEV1%, FEV1/FVC, blood eosinophil count, FeNO, smoking history, allergic rhinitis, CRSsNP, CRSwNP, and enrolled trial. Results pooled across 10 multiply imputed datasets using Rubin's rules. aRR: Adjusted rate ratio from negative binomial regression. IQR (Interquartile Range): Rate ratio per increase from 25th to 75th percentile of ACQ-5. Full ORACLE2 IQR**:** 1.200 (25th: 1.800, 75th: 3.000). Any ACQ Subset IQR: 1.400 (25th: 0.600, 75th: 2.000).

TABLE S9.ACQ-5 Increment Needed for Target Rate Ratios

Cohort	Model	ACQ increment for aRR = 1.2	ACQ increment for aRR = 1.4
Full ORACLE2	Univariable*	0.768 [0.629-0.985]	1.417 [1.161-1.818]
Full ORACLE2	Multivariable**	1.055 [0.813-1.502]	1.946 [1.500-2.771]
Any ACQ Subset	Univariable*	0.815 [0.511-2.007]	1.505 [0.944-3.705]
Any ACQ Subset	Multivariable**	1.102 [0.615-5.318]	2.033 [1.134-9.815]

Values indicate how much ACQ-5 needs to increase to achieve the target adjusted rate ratio (aRR) for exacerbations. For example, if ACQ increment for aRR = 1.2 is 0.5, this means increasing ACQ-5 by 0.5 points is associated with a 20% increase in exacerbation rate. *Univariable covariates: ACQ + trial adjustment only. **Multivariable covariates: adjusted for ACQ-5, sex, BMI, severe attack history in preceding 12 months (yes/no), GINA treatment step, FEV1%, FEV1/FVC, blood eosinophil count, FeNO, smoking history, allergic rhinitis, CRSsNP, CRSwNP, and enrolled trial. Results pooled across 10 multiply imputed datasets using Rubin's rules. Values shown as point estimate [95% CI]

TABLE S10. Adjusted rate ratios for ACQ-5 ordinal classification

ACQ-5 Category	Full ORACLE2 (n=6513)			Any ACQ Subset (n=750)		
	n (%)	aRR [95% CI]	p-value	n (%)	aRR [95% CI]	p-value
<0.75 (REF)	351 (5.4%)	1.0 [REF]	-	233 (31.1%)	1.0 [REF]	-
0.75 - <1.5 vs <0.75	617 (9.5%)	1.273 [0.909 - 1.782]	0.1594	225 (30.0%)	1.306 [0.833 - 2.047]	0.2439
1.5 - <2.0 vs <0.75	761 (11.7%)	1.350 [0.966 - 1.884]	0.0784	81 (10.8%)	1.227 [0.693 - 2.173]	0.4827
2.0 - <2.5 vs <0.75	1629 (25.0%)	1.347 [0.978 - 1.855]	0.0683	90 (12.0%)	1.066 [0.633 - 1.796]	0.8096
2.5 - <3.0 vs <0.75	1092 (16.8%)	1.442 [1.042 - 1.995]	0.0274	41 (5.5%)	1.063 [0.585 - 1.930]	0.8418
>=3.0 vs <0.75	2063 (31.7%)	1.781 [1.295 - 2.450]	0.0004	80 (10.7%)	1.957 [1.189 - 3.222]	0.0084

Multivariable covariates: adjusted for ACQ-5, sex, BMI, severe attack history in preceding 12 months (yes/no), GINA treatment step, FEV1%, FEV1/FVC, blood eosinophil count, FeNO, smoking history, allergic rhinitis, CRSsNP, CRSwNP, and enrolled trial. Results pooled across 10 multiply imputed datasets using Rubin's rules. Values shown as adjusted rate ratio (aRR) and [95% CI].

TABLE S11 – Asthma attack prediction model performance

Model	Explained variance (R^2)	Mean AIC	E/O ratio	Calibration slope
Model 1: Full model* (Reference)	0.44	13701	0.993	0.988
Model 2: ACQ Only	0.18	14063	0.908	1.108
Model 3: Full without ACQ	0.42	13743	0.992	1.008
Δ (Model 3 vs 1)	-0.02	+42	-0.001	+0.020
Model 4: Attack history only	0.21	14081	0.936	1.109
Model 5: Full without attack history	0.38	13750	0.984	1.012
Δ (Model 5 vs 1)	-0.06	+49	-0.009	+0.024
Model 6: Biomarkers only (blood eos×FeNO)	0.18	14023	0.889	1.014
Model 7: Full without biomarkers	0.40	13818	0.988	0.989
Δ (Model 7 vs 1)	-0.04	+117	-0.005	+0.001

*Full model adjusted for: ACQ-5 mean score, sex, BMI, severe attack history in preceding 12 months (yes/no), GINA treatment step, FEV1%, FEV1/FVC, blood eosinophil (eos) count, FeNO, blood eosinophil count * FeNO, smoking history, allergic rhinitis, CRSsNP, CRSwNP. E/O ratio = expected / observed ratio.

3. Supplementary References

1 Brusselle GG, VanderStichele C, Jordens P, *et al.* Azithromycin for prevention of exacerbations in severe asthma (AZISAST): A multicentre randomised double-blind placebo-controlled trial. *Thorax* 2013; **68**: 322–9.

2 Castro M, Wenzel SE, Bleecker ER, *et al.* Benralizumab, an anti-interleukin 5 receptor α monoclonal antibody, versus placebo for uncontrolled eosinophilic asthma: A phase 2b randomised dose-ranging study. *Lancet Respir Med* 2014; **2**: 879–90.

3 Lee LA, Bailes Z, Barnes N, *et al.* Efficacy and safety of once-daily single-inhaler triple therapy (FF/UMEC/VI) versus FF/VI in patients with inadequately controlled asthma (CAPTAIN): a double-blind, randomised, phase 3A trial. *Lancet Respir Med* 2021; **9**: 69–84.

4 Harris JM, Maciuca R, Bradley MS, *et al.* A randomized trial of the efficacy and safety of quilizumab in adults with inadequately controlled allergic asthma. *Respir Res* 2016; **17**: 1–11.

5 Pavord ID, Korn S, Howarth P, *et al.* Mepolizumab for severe eosinophilic asthma (DREAM): A multicentre, double-blind, placebo-controlled trial. *The Lancet* 2012; **380**: 651–9.

6 Wenzel S, Castro M, Corren J, *et al.* Dupilumab efficacy and safety in adults with uncontrolled persistent asthma despite use of medium-to-high-dose inhaled corticosteroids plus a long-acting β2 agonist: a randomised double-blind placebo-controlled pivotal phase 2b dose-ranging trial. *The Lancet* 2016; **388**: 31–44.

7 Hanania NA, Wenzel S, Roseń K, *et al.* Exploring the effects of omalizumab in allergic asthma: An analysis of biomarkers in the EXTRA study. *Am J Respir Crit Care Med* 2013; **187**: 804–11.

8 Hanania NA, Korenblat P, Chapman KR, *et al.* Efficacy and safety of lebrikizumab in patients with uncontrolled asthma (LAVOLTA I and LAVOLTA II): replicate, phase 3, randomised, double-blind, placebo-controlled trials. *Lancet Respir Med* 2016; **4**: 781–96.

9 Brightling CE, Gaga M, Inoue H, *et al.* Effectiveness of fevipiprant in reducing exacerbations in patients with severe asthma (LUSTER-1 and LUSTER-2): two phase 3 randomised controlled trials. *Lancet Respir Med* 2021; **9**: 43–56.

10 Hanania NA, Noonan M, Corren J, *et al.* Lebrikizumab in moderate-to-severe asthma: Pooled data from two randomised placebo-controlled studies. *Thorax* 2015; **70**: 748–56.

11 Corren J, Lemanske RF, Hanania NA, *et al.* Lebrikizumab treatment in adults with asthma. *New England Journal of Medicine* 2011; **365**: 1088–98.

12 Menzies-Gow A, Corren J, Bourdin A, *et al.* Tezepelumab in Adults and Adolescents with Severe, Uncontrolled Asthma. *New England Journal of Medicine* 2021; **384**: 1800–9.

13 Beasley R, Holliday M, Reddel HK, *et al.* Controlled trial of budesonide-formoterol as needed for mild asthma. *New England Journal of Medicine* 2019; **380**: 2020–30.

14 Sorkness CA, Lemanske RF, Mauger DT, *et al.* Long-term comparison of 3 controller regimens for mild-moderate persistent childhood asthma: The Pediatric Asthma Controller Trial. *Journal of Allergy and Clinical Immunology* 2007; **119**: 64–72.

15 Corren J, Parnes JR, Wang L, *et al.* Tezepelumab in Adults with Uncontrolled Asthma. *New England Journal of Medicine* 2017; **377**: 936–46.

16 Hardy J, Baggott C, Fingleton J, *et al.* Budesonide-formoterol reliever therapy versus maintenance budesonide plus terbutaline reliever therapy in adults with mild to moderate

asthma (PRACTICAL): a 52-week, open-label, multicentre, superiority, randomised controlled trial. *Lancet* 2019; **394**: 919–28.

17 Castro M, Corren J, Pavord ID, *et al.* Dupilumab Efficacy and Safety in Moderate-to-Severe Uncontrolled Asthma. *New England Journal of Medicine* 2018; **378**: 2486–96.

18 Panettieri RA, Sjöbring U, Péterffy AM, *et al.* Tralokinumab for severe, uncontrolled asthma (STRATOS 1 and STRATOS 2): two randomised, double-blind, placebo-controlled, phase 3 clinical trials. *Lancet Respir Med* 2018; **6**: 511–25.

19 Meulmeester FL, Mailhot-Larouche S, Celis-Preciado C, *et al.* Inflammatory and clinical risk factors for asthma attacks (ORACLE2): a patient-level meta-analysis of control groups of 22 randomised trials. *Lancet Respir Med* 2025.

20 Celis-Preciado CA, Leclerc S, Duval M, *et al.* Phenotyping the Responses to Systemic Corticosteroids in the Management of Asthma Attacks (PRISMA): protocol for an observational and translational pilot study. *BMJ Open Respir Res* 2023; **10**: e001932.

21 Howell I, Mahdi M, Bafadhel M, *et al.* Recovery of Breakthrough Asthma Attacks Treated With Oral Steroids While on Monoclonal Antibody Therapy: Protocol for a Prospective Observational Study (BOOST). *JMIR Res Protoc* 2023; **12**: e46741.

22 Couillard S, Shrimanker R, Chaudhuri R, *et al.* Fractional Exhaled Nitric Oxide Nonsuppression Identifies Corticosteroid-Resistant Type 2 Signaling in Severe Asthma. *Am J Respir Crit Care Med* 2021; **204**: 731–4.
